# The core interaction network of transcription elongation factors is broadly maintained across diverse bacteria

**DOI:** 10.1128/mbio.02180-26

**Published:** 2026-10-23

**Authors:** Thao N. P. Tran, Reid E. Peterson, Xiaofang Jiang, Swathi A. Sevugan, Sandeepani Ranaweera, Paul Babitzke, David Fushman, Wade C. Winkler

**Affiliations:** 1 Department of Chemistry and Biochemistry, Center for Biomolecular Structure and Organization, University of Maryland 1068 , College Park, Maryland, USA; 2 Department of Cell Biology and Molecular Genetics, University of Maryland 1068 , College Park, Maryland, USA; 3 Intramural Research Program, National Library of Medicine, National Institutes of Health 2511, Bethesda, Maryland, USA; 4 Department of Biochemistry and Molecular Biology, Center for RNA Molecular Biology, Pennsylvania State University 189499, University Park, Pennsylvania, USA; University of California, Berkeley, Berkeley, California, USA

**Keywords:** transcription elongation, antitermination, transcription-translation coupling, *Bacillus subtilis*

## Abstract

Bacterial transcription elongation has been investigated most thoroughly for the model bacterium *Escherichia coli.* The *E. coli* elongation factor NusG associates through its N-terminal domain with the transcription elongation complex (TEC), while its C-terminal domain forms mutually exclusive interactions with either ribosomal protein S10 or Rho termination factor. The *E. coli* NusG:S10 complex bridges the TEC with a pioneering ribosome for coupling of transcription and translation. NusG also participates in antitermination complexes (ATC) that inhibit Rho-dependent termination and increase transcription of rRNA operons. A characteristic sequence in the nascent *rrn* RNA, called *boxA*, loads *E. coli* NusB:S10 onto the NusG-bound TEC, leading to the formation of the *rrn* ATC. However, these findings may not extend to all bacteria. Rho-dependent termination is less important in gram-positive bacteria, including some that do not encode Rho, and transcription and translation remain uncoupled *in Bacillus subtilis* and many other gram-positive bacteria. It is therefore unclear whether the interaction network for *E. coli* elongation factors is conserved in these organisms. We identified *boxA*-like sequences upstream of 16S and 23S genes in *Bacillus* species and found that they associate directly with NusB. We also found that *B. subtilis* S10 associates with both NusB and NusG, and that Rho can also associate with NusG. Bioinformatic analyses revealed conservation of interaction interface residues across multiple phyla. Together, these data demonstrate that the core interaction network of bacterial elongation factors is broadly conserved across diverse bacteria, despite differences in how these interactions function in individual species.

**IMPORTANCE:**

*Escherichia coli* is known to use several key elongation factors that promote direct coupling of transcription with translation. These factors also trigger the formation of an antitermination complex that protects the synthesis of ribosomal RNA and CRISPR transcripts from interference by the Rho termination factor. However, *Bacillus subtilis* is not thought to couple transcription with translation. It is also not known whether *B. subtilis* needs to suppress Rho, given that its Rho is dispensable. Therefore, we purified *B. subtilis* elongation factors and mapped their interaction network. Surprisingly, we find that the *B. subtilis* elongation factors form complexes that closely resemble *E. coli* counterparts, despite the significant mechanistic differences in how these bacteria control transcription elongation.

## INTRODUCTION

Early electron micrographs of *Escherichia coli* suggested that a lead ribosome might be located directly adjacent to transcribing RNA polymerase (1, 2). It was later demonstrated that the presence of this ribosome, sometimes called the pioneering or lead ribosome, reduces premature transcription termination and inhibits the backtracking of the transcription elongation complex (TEC) (3–7). Correspondingly, antibiotics affecting transcription impact both transcription and translation rates (8), and disruption of transcription-translation coupling causes premature transcription termination (9). Furthermore, the *in vivo* rates of *E. coli* transcription and translation are empirically matched (10, 11). Together, these observations suggest that the *E. coli* TEC is purposefully coupled to a pioneering ribosome (3), a hypothesis that has gained support from recent high-resolution structural data (12–17).

**Fig 1:**
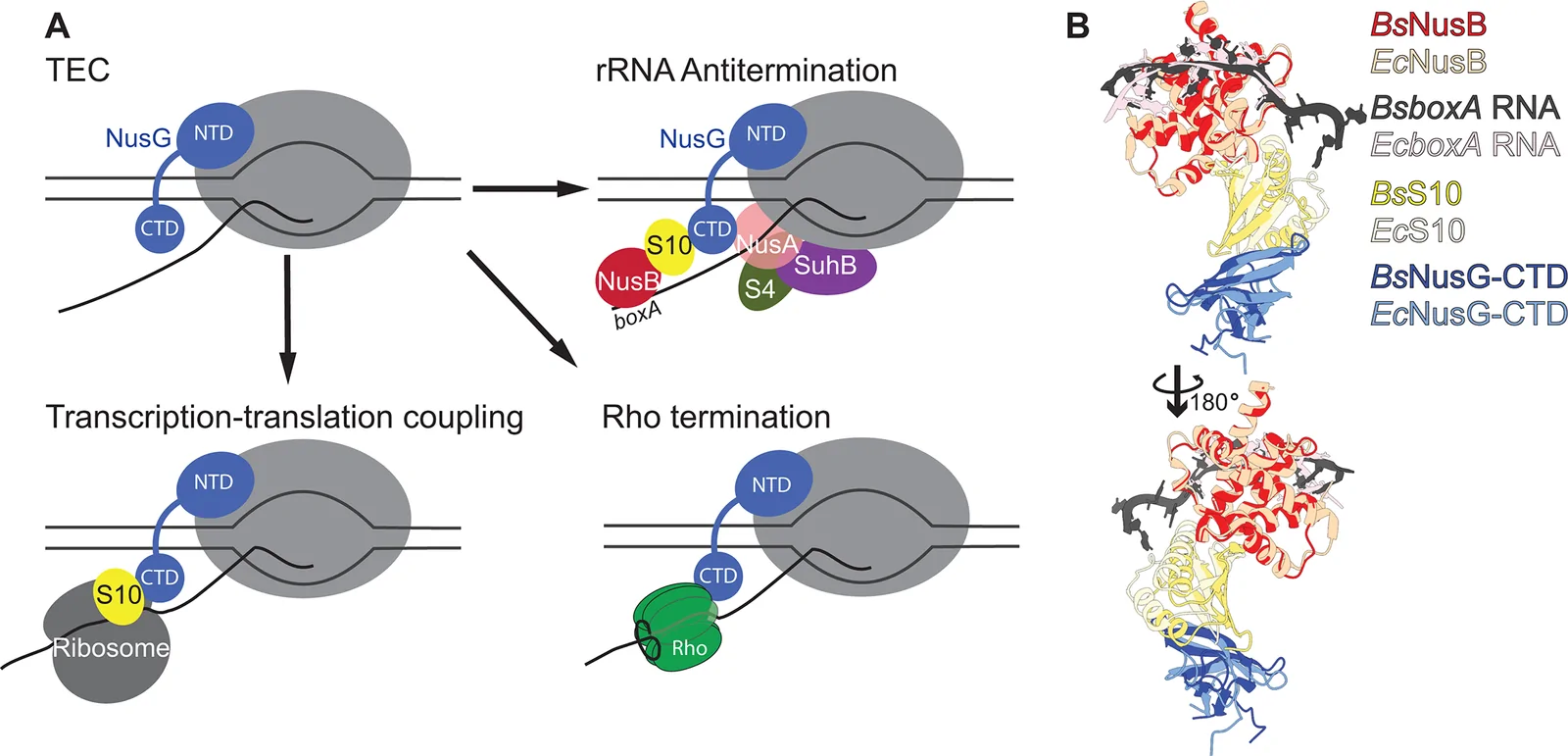
Interactions among transcription elongation factors. (**A**) Cartoon schematics summarize the key interactions exhibited by *E. coli* transcription elongation factors, as discussed in the text. (**B**) Comparison of the *Ec*NusB:S10:*boxA* complex (PDB: 3D3C) with the predicted structure of *Bs*NusB:S10:*boxA*, as predicted by AlphaFold 3. Similarly, this structural model includes an overlay of the *Ec*NusG-CTD:S10 complex (PDB: 2KVQ) with the *Bs*NusG-CTD:S10 complex, as predicted by AlphaFold 3. The intermolecular interfaces exhibited by the *Bs*NusB:*boxA*, *Bs*NusB:S10, and *Bs*S10:NusG complexes generally resemble the *E. coli* counterparts, suggesting that the *E. coli* interaction network is maintained in *B. subtilis*, despite the lack of transcription-translation coupling and despite the lack of direct evidence for rRNA antitermination.

The transcription and translation machinery are specifically bridged by an interaction between elongation factor NusG from the TEC and the S10 protein from the ribosome. NusG associates with the TEC via its N-terminal domain (NTD) (18, 19), while its C-terminal domain (CTD) partners in a mutually exclusive manner with free S10 (20), S10-associated ribosomes (21, 22), or Rho termination factor (23–29). A model for *E. coli* gene expression has emerged from these observations: the TEC is coupled to a pioneering ribosome during active translation, thereby inhibiting Rho termination; however, when S10 is not nearby, or when transcription-translation coupling is disrupted, Rho interaction with the TEC, which can be strengthened by NusG, can increase transcription termination (Fig. 1A) (20, 30).

NusG’s involvement in *E. coli* gene expression does not end with transcription-translation coupling. While the NusG:S10 complex links transcription with translation, the mutually exclusive NusG:Rho complex serves to enhance termination (31, 32). This implies that Rho could theoretically interfere with the synthesis of any noncoding RNA, such as rRNA operons and CRISPR RNA arrays, given that transcription-translation coupling would be absent at those genomic loci. However, to suppress Rho-dependent termination, *E. coli* employs processive antitermination, a mechanistic strategy that occurs when the TEC is modified to resist termination over long distances (33). A specific antitermination complex (ATC) has been shown to protect rRNA operons (*rrn*) from Rho-dependent termination (34, 35) (Fig. 1A). In the *rrn* ATC, a metabolic protein, SuhB, binds the TEC along with ribosomal protein S10, S4, and the elongation factors NusA, NusG, and NusB (36). In this complex, instead of binding ribosome-associated S10, the NusG-CTD associates with an S10-NusB complex (37), which is recruited to the TEC by its association with a short RNA sequence in the nascent transcript called *boxA* (38–40). The *boxA* RNA sequence is essential for the *rrn* ATC and its presence is sufficient to prompt ATC formation (41). Structural data show that the *rrn* ATC moves NusA away from the RNA exit tunnel to interfere with intrinsic termination, while also sterically blocking Rho from accessing the TEC (42, 43). Because of these outcomes, the *rrn* ATC is important for ribosomal maturation and *E. coli* physiology (36). However, there are many fundamental differences in the mechanisms of transcription elongation between gram-negative and gram-positive bacteria.

Unlike *E. coli*, *Bacillus subtilis* is not believed to couple transcription with translation (10, 44), and unlike *E. coli*, *B. subtilis* Rho and NusG are dispensable (45, 46). Together, these observations suggest that *B. subtilis* might not require an ATC to protect the synthesis of noncoding RNAs such as rRNAs. Therefore, the protein-protein and protein-RNA interactions exhibited by *E. coli* elongation factors might not occur with their *B. subtilis* counterparts. To investigate this possibility, we purified *B. subtilis* elongation factors *Bs*NusG, *Bs*NusB, *Bs*Rho, and *Bs*S10 and investigated their interactions. Our data conclusively demonstrate that the *B. subtilis* elongation factors form an interaction network similar to that of *E. coli*. Specifically, *Bs*NusB associates with *Bs*S10, which associates with *Bs*NusG, which can associate with *Bs*Rho in a mutually exclusive manner. Also, we identified a *boxA*-like RNA sequence that is conserved upstream of the *B. subtilis rrn* operons and found that it associates directly with *Bs*NusB. Therefore, despite their fundamental differences in utilization of Rho and in the coupling of transcription with translation, *B. subtilis* and *E. coli* still both rely on the same core protein-protein interaction network among their transcription elongation factors.

## RESULTS

### *B. subtilis* NusB associates with a *boxA*-like RNA sequence

Formation of the *E. coli rrn* ATC starts with the association of a NusB:S10 complex with the *boxA* RNA sequence (38). *BoxA* is located within the leader region of the *E. coli rrn* operon, positioned upstream of the 16S and 23S genes (41). *boxA* is also located upstream of other gammaproteobacterial genes, such as *suhB* and CRISPR operons (39, 43). It was previously noted that *B. subtilis rrn* operons might begin with a small hairpin at the 5′ terminus and also feature *boxA*-like elements upstream of 16S (41). Moreover, addition of a *B. subtilis* region that included a *boxA*-like sequence could restore activity of an *rrn* antitermination reporter in *E. coli* (47). However, while *E. coli rrn* antitermination has been reconstituted *in vitro* (34, 42), the possibility of a *B. subtilis rrn* ATC has not been experimentally investigated. Therefore, we examined an alignment of leader regions for the *B. subtilis rrn* operons (Fig. S1). It was previously predicted that *B. subtilis rrn* operons might begin with a small hairpin at the 5′ terminus (41). Indeed, we herein note the presence of a small hairpin in the *B. subtilis rrn* operons (Fig. S1). Immediately downstream of this helix is a well-conserved *boxA*-like sequence, although its consensus (AUGAUCUUUGAAA) differs from the *E. coli* consensus (UGCUCUUUAACA).

**Fig 2:**
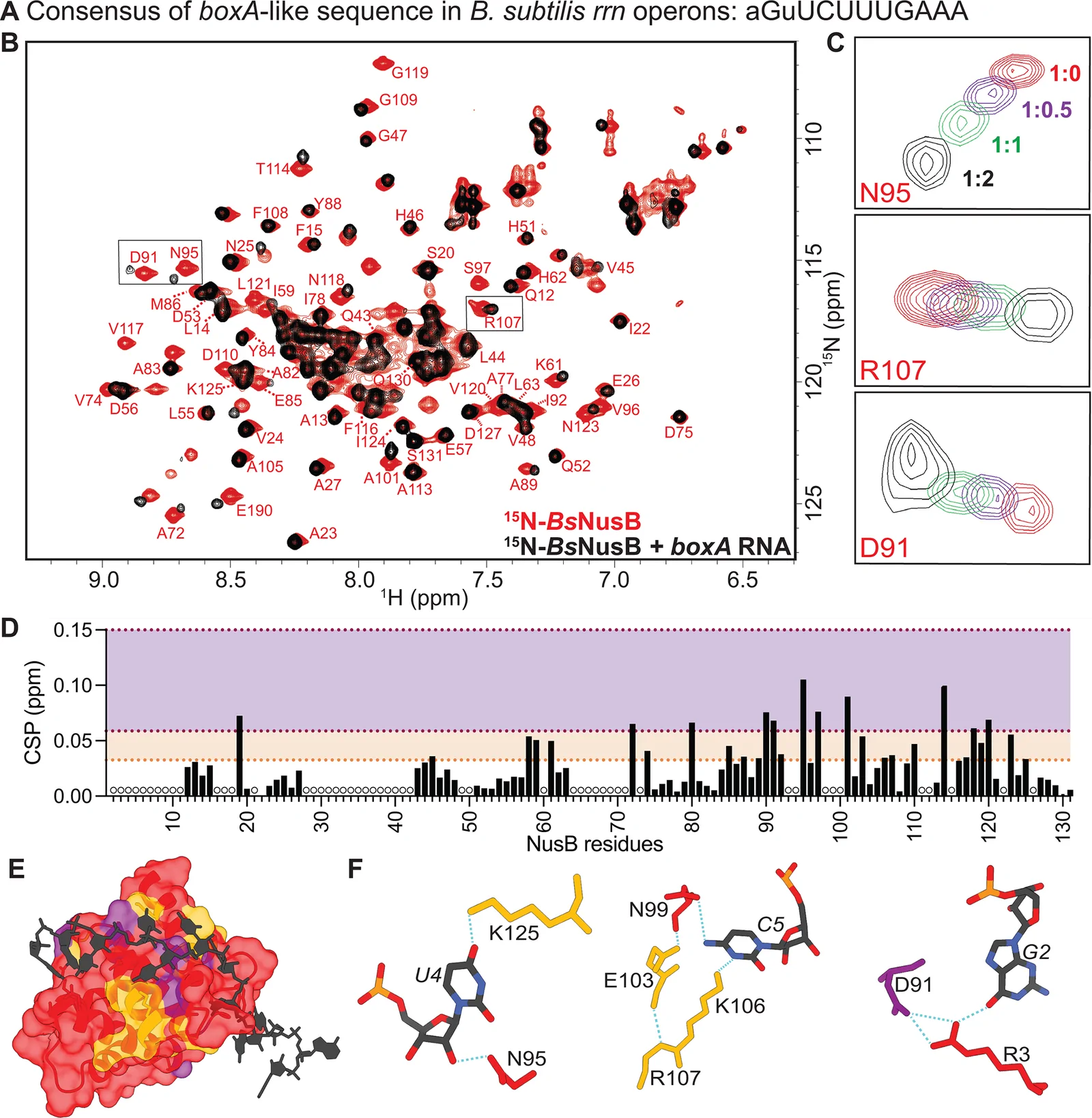
*B. subtilis* NusB associates with a *boxA*-like RNA. (**A**) A multiple sequence alignment of *B. subtilis rrn* operons (Fig. S1) revealed a *boxA*-like sequence with the consensus shown in this panel. (**B**) Overlay of ^1^H-^15^N HMQC spectra of ^15^N-labeled *Bs*NusB free in solution at 200 μM (red) and in the presence of two molar equivalents of unlabeled *boxA* RNA (black). (**C**) Unlabeled *boxA* RNA was titrated into ^15^N-labeled *Bs*NusB with a molar ratio of 0.5× (purple), 1× (green), and 2× (black). Selected *Bs*NusB peaks that exhibited significant shifts are displayed. (**D**) Chemical shift perturbations (CSPs) for all assigned residue peaks of *Bs*NusB determined using equation 1. Some residues were not assigned to specific peaks (indicated by open circles). Orange shading indicates CSP values above the average CSP. Purple shading indicates CSP values that are greater than 1 standard deviation above the average. (**E**) AlphaFold 3 model of the *Bs*NusB:*boxA* RNA complex showing locations of NusB residues that exhibited amide signal shifts either above the average CSP (orange) or more than 1 standard deviation above the average CSP (purple). (**F**) Representative NusB residues that are predicted to interact with RNA and that exhibited higher-than-average CSP are shown with putative hydrogen-bonding interactions (blue dashed lines).

While high-resolution structural data have been obtained for the *Ec*NusB:*boxA* complex (40), the corresponding *B. subtilis* factors have not been structurally examined. Therefore, we predicted the structure of *B. subtilis* factors using AlphaFold 3 (48). This analysis revealed a high degree of structural similarity between *E. coli* and *B. subtilis* elongation factors (Fig. 1B). To experimentally investigate this possibility, we expressed and purified *Bs*NusB (Fig. S2 and S3) and used nuclear magnetic resonance (NMR) to investigate whether it associates directly with the *rrnO boxA* RNA. Specifically, we purified ^15^N-labeled *Bs*NusB and titrated it with unlabeled *box*A RNA while monitoring the change in *Bs*NusB ^1^H and ^15^N chemical shifts using 2D ^1^H-^15^N heteronuclear multiple quantum coherence (HMQC) experiments. *BoxA* RNA was titrated in 0.5×, 1×, and 2× molar ratios. The addition of *boxA* RNA resulted in shifts of specific *Bs*NusB signals (Fig. 2A through D), indicating that *boxA* binds to the protein. A majority of the perturbed signals exhibited gradual shifts with a traceable trajectory, consistent with a fast-exchange binding regime.

To determine if a site-specific binding interface exists between *Bs*NusB and *boxA*, we expressed and purified ^15^N/^13^C-labeled *Bs*NusB and titrated *boxA* to study their interaction. We achieved a mostly complete assignment of NusB ^1^H and ^15^N backbone amide chemical shifts of the observable signals (Fig. S1). Amide chemical shift perturbations (CSPs) in *Bs*NusB caused by *boxA* were analyzed (see equation 1) to identify and map significantly affected residues (Fig. 2C through F). While the majority of CSPs were rather small (average observable CSP = 0.033 ppm), residues with signal perturbations more than 1 standard deviation above the average CSP were localized primarily across C-terminal α-helices of *Bs*NusB.

The AlphaFold 3 analysis produced a ribonucleoprotein complex that closely resembled the corresponding *E. coli* complex (Fig. S5) and agreed with the significant CSPs observed in our NMR titration (Fig. 2D through F). For example, examination of the *Bs*NusB electrostatic surface potential revealed a primarily electronegative surface, with a few small electropositive patches that are predicted to interact with RNA and that involve residues that exhibit significant CSPs (Fig. S5). Additionally, several *Bs*NusB amino acids that are predicted to directly interact with either nucleobases or their associated ribose (Fig. S5) also exhibit significant CSPs (Fig. 2E and F). Together, these biochemical and biophysical data demonstrate that *Bs*NusB directly associates with the *boxA* RNA identified herein, similar to the *E. coli* determinants that are used by Nus factor antitermination complexes.

### *B. subtilis* NusB associates with S10

**Fig 3:**
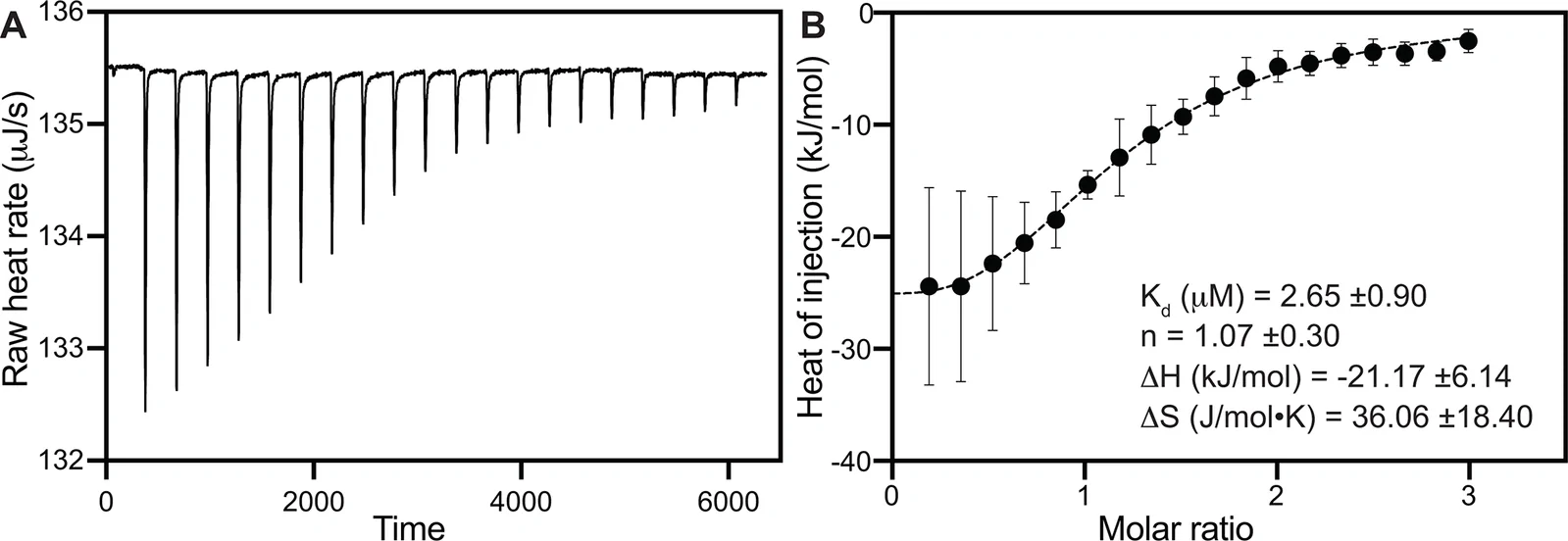
Isothermal titration calorimetry analysis of *B. subtilis* NusB and S10. (**A**) A representative raw thermogram of S10 titrated into NusB with background S10 dilution subtracted. (**B**) Integrated heats of injection (circular dots) plotted against molar ratio of S10 to NusB, fitted to a one-site binding model (dashed line). The dissociation constant (*K*_*d*_), enthalpy change (Δ*H*), stoichiometry (*n*), and entropy change (Δ*S*) are derived from the fit of three independent experiments (errors represent SD).

**Fig 4:**
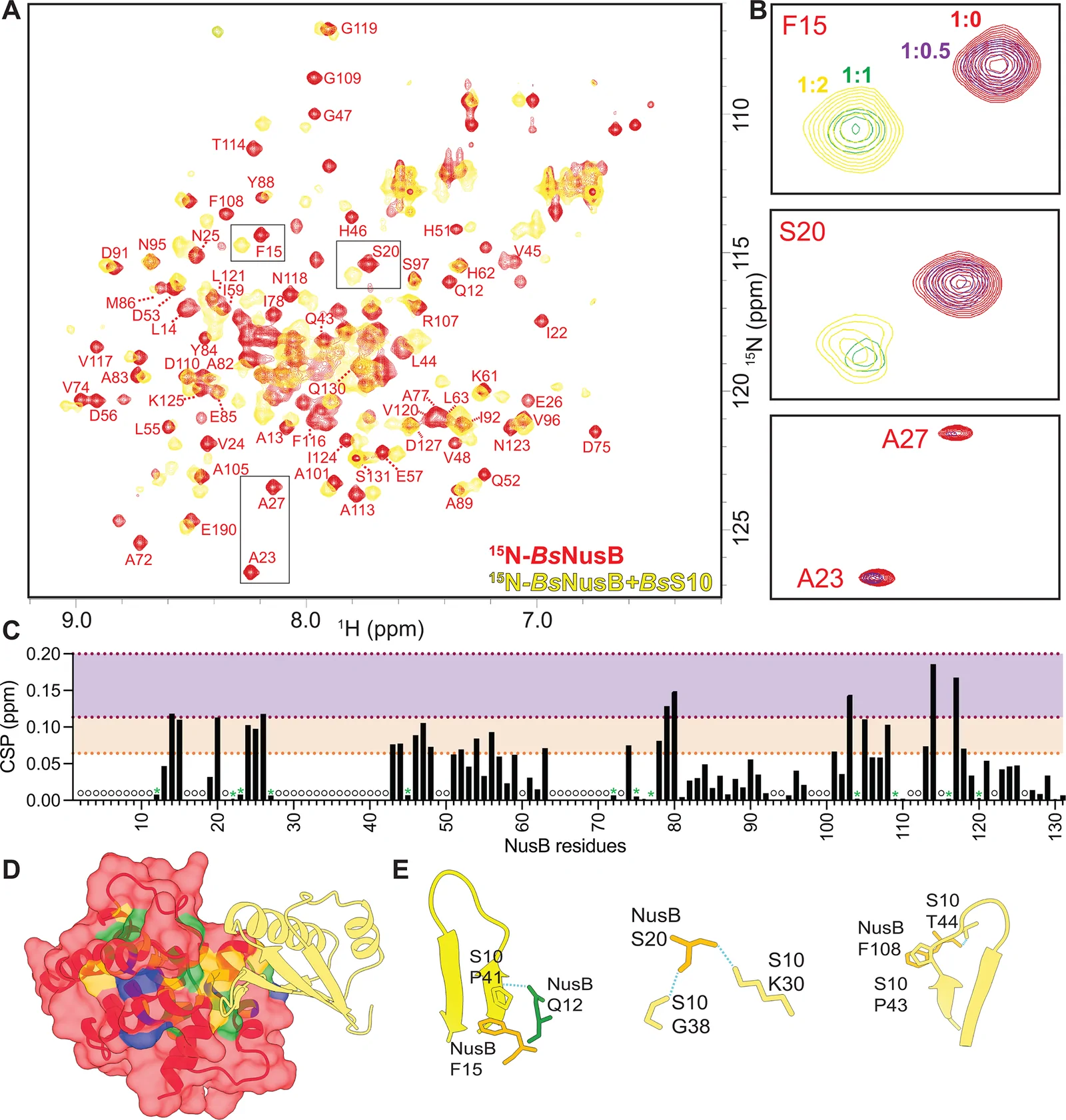
*B. subtilis* NusB forms a complex with S10. (**A**) Overlay of ^1^H-^15^N HMQC spectra of ^15^N-labeled *Bs*NusB free in solution at 200 μM (red) and in the presence of two molar equivalents of unlabeled S10 (yellow). (**B**) Unlabeled S10 was titrated into ^15^N-labeled *Bs*NusB with a molar ratio of 0.5× (purple), 1× (green), and 2× (yellow). Selected *Bs*NusB peaks that shifted by more than 1 standard deviation above the average CSP or were strongly attenuated are displayed. (**C**) Amide CSPs for *Bs*NusB determined as described in Fig. 1D. Green asterisks indicate residues that were assigned but whose corresponding peaks were strongly attenuated upon addition of S10. (**D**) AlphaFold 3 model of the *Bs*NusB:*Bs*S10 complex, showing locations of NusB residues with signal shifts either above the average CSP (orange), or more than 1 standard deviation above the average CSP (purple), or exhibiting strong signal attenuation (green). (**E**) Representative residues from the NusB:S10 interface are shown with putative hydrogen-bonding interactions (blue dashed lines).

The *Ec*NusB:S10 complex is stably maintained in *E. coli,* and *Ec*NusB is therefore likely to compete against the ribosome for binding S10. However, it has not been directly investigated whether *B. subtilis* NusB associates with S10. To that end, we expressed and purified *B. subtilis* NusB and S10 (Fig. S2). An isothermal titration calorimetry (ITC) analysis revealed that *Bs*S10 associates with *Bs*NusB (Fig. 3A and B; Fig. S3) with a *K*_*d*_ of 2.65 ± 0.9 µM. To examine the *Bs*NusB:*Bs*S10 interaction further, we employed NMR analysis of ^15^N-labeled *Bs*NusB with titration of *Bs*S10 (Fig. 4A and B). Amide ^1^H and ^15^N signal shifts were analyzed and plotted on a CSP graph (Fig. 4C). Unlike *Bs*NusB signals that gradually shifted with the addition of *boxA*, practically all perturbed *Bs*NusB signals experienced full attenuation accompanied by the appearance of a new nearby signal, presumably corresponding to the bound state of the same residue. This behavior is a hallmark of a slow-exchange binding regime (49, 50), which suggests a tight or moderate interaction (slow dissociation rate) between *Bs*NusB and *Bs*S10. This slow-exchange binding agrees with the low-micromolar binding affinity measured by ITC.

An AlphaFold 3 model of the *Bs*NusB:*Bs*S10 complex resembled the corresponding *E. coli* complex (Fig. S1). Several significantly perturbed *Bs*NusB residues are located at the putative interface, such as F15 and F108 (Fig. 4D and E; Fig. S6), which are predicted to stack with *Bs*S10 prolines P41 and P43. There are also significantly perturbed residues that are not at the putative interface but are instead predicted to be located within the core of *Bs*NusB, such as residues 46–48 and 77–80. Signal perturbation of these solvent-inaccessible residues suggests that an intramolecular rearrangement of *Bs*NusB is either required for binding of *Bs*S10 or is triggered by binding of *Bs*S10.

Based on these data and the analysis of sequence alignments between *Ec*NusB and *Bs*NusB, we introduced Q12A and S20L mutations into *Bs*NusB, as the corresponding *Ec*NusB residues were previously reported to be involved in the interaction with *Ec*S10 (51) (Fig. 4E). We expressed and purified the mutant protein and subjected it to analysis by circular dichroism, which revealed no significant changes in the secondary structure composition (Fig. S3). ITC analysis of Q12A/S20L *Bs*NusB titrated with *Bs*S10 (Fig. S3) revealed a weaker equilibrium binding affinity, consistent with a role in mediating protein-protein interactions.

Together, these data demonstrate that *B. subtilis* NusB and S10 form a complex. This complex is likely to closely resemble its *E. coli* counterpart, which, in *E. coli*, is specifically used in Nus factor antitermination complexes.

### *B. subtilis* NusB associates with NusG

**Fig 5:**
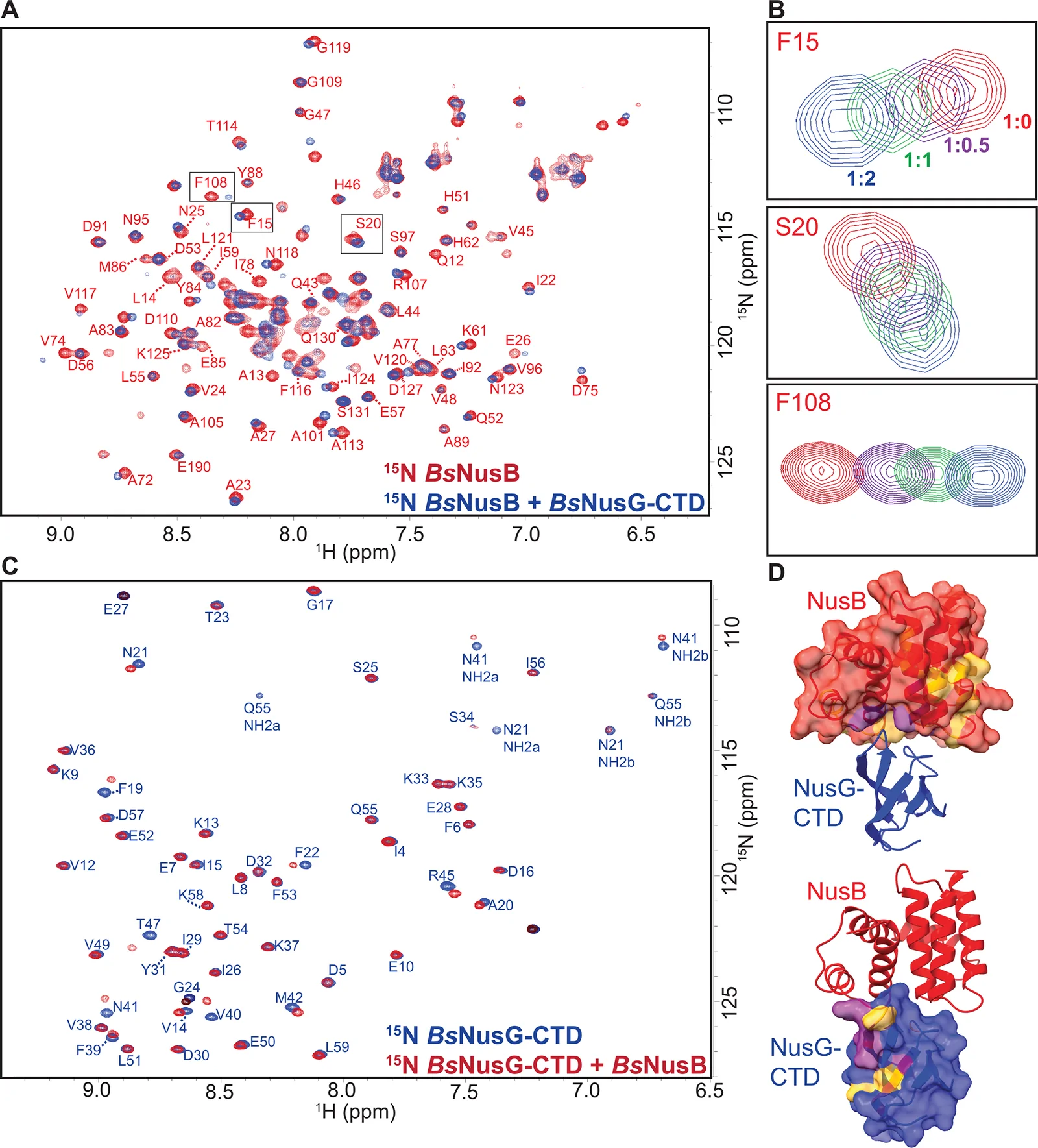
*B. subtilis* NusB associates with NusG. (**A**) Overlay of ^1^H-^15^N HMQC spectra of ^15^N-labeled *Bs*NusB free in solution at 200 μM (red) and in the presence of two molar equivalents of unlabeled NusG-CTD (blue). (**B**) Unlabeled NusG-CTD was titrated into ^15^N-labeled *Bs*NusB with a molar ratio of 0.5× (purple), 1× (green), and 2× (blue). Selected *Bs*NusB peaks that shifted by more than 1 standard deviation above the average CSP are displayed. (**C**) Overlay of ^1^H-^15^N HMQC spectra of ^15^N-labeled *Bs*NusG-CTD free in solution at 200 μM (blue) and in the presence of two molar equivalents of unlabeled NusB (red). (**D**) AlphaFold 3 model of the *Bs*NusB:*Bs*NusG complex, showing in the top panel the locations of NusB residues with signal shifts either above the average CSP (orange) or more than 1 standard deviation above the average CSP (purple). The lower panel shows locations of NusG-CTD residues that exhibited significant CSPs.

Prior NMR studies did not observe a complex between *Ec*NusB and *Ec*NusG (20). However, under conditions similar to those previously used to investigate the *E. coli* proteins, we observed an interaction between *Bs*NusB and *Bs*NusG (Fig. 5A; Fig. S7). We incubated ^15^N-labeled *Bs*NusB with either full-length *Bs*NusG (Fig. S7) or BsNusG-CTD (Fig. 5A), separately, using molar ratio increments similar to those used above. When these *Bs*NusG proteins were titrated into ^15^N-labeled *Bs*NusB, most, if not all, *Bs*NusB residues that were perturbed by the addition of full-length *Bs*NusG were also perturbed to the same degree by *Bs*NusG-CTD (Fig. 5A; Fig. S7). All affected *Bs*NusB signals experienced traceable shifts consistent with a fast-exchange binding regime and a generally weaker interaction. By contrast, a mixture of ^15^N-labeled *Bs*NusB and isolated *Bs*NusG-NTD did not result in substantial NMR signal shifts that could suggest a meaningful interaction (Fig. S8). Together, these data indicate that the NusB:NusG complex specifically involves the NusG-CTD.

We then determined a dissociation constant (*K*_*d*_) of the *Bs*NusB:*Bs*NusG-CTD complex (Fig. S7). Unlike previous titrations, where ligand was capped at a 2× molar ratio for the purpose of qualitative analysis of perturbed residues, it was deemed necessary to reach full saturation, wherein practically all ^15^N-labeled *Bs*NusB molecules would exist in a bound state. Therefore, *Bs*NusG-CTD was titrated up to a 10× molar ratio until no further change in ^15^N-labeled *Bs*NusB chemical shifts was detected. Chemical shifts were analyzed at each titration point, and the corresponding CSPs were globally fit to determine the dissociation constant using an in-house MATLAB program Kdfit (52). We found this interaction to have a *K*_*d*_ of ~375 ± 65 µM (Fig. S7).

We also performed a reverse titration by monitoring changes in NMR spectra of ^15^N-labeled *Bs*NusG-CTD upon addition of unlabeled *Bs*NusB. To determine which NMR signals correspond to each amino acid residue, the unbound ^15^N-labeled *Bs*NusG-CTD protein was used to obtain a near-complete ^1^H and ^15^N backbone amide chemical shift assignment (Fig. 5C; Fig. S9). We observed a similar fast-exchange binding regime and generally weak, yet specific interactions between ^15^N-labeled *Bs*NusG-CTD and *Bs*NusB (in contrast to the lack of substantial NMR signal shifts of ^15^N-labeled *Bs*NusG-CTD upon addition of *Bs*NusG-NTD in Fig. S8). There are no prior structural data on a NusB:NusG complex; therefore, we surveyed a putative model predicted by AlphaFold 3 (Fig. 5D). This model identified a subset of *Bs*NusG-CTD residues with significant CSPs that correlated with the putative NusB:NusG interface. Importantly, this portion of NusB also corresponds to the region that is predicted to associate with S10. For example, all NusB residues that exhibited NMR signal shifts and/or strong attenuations are likely involved in the *Bs*NusB:*Bs*S10 interaction also showed signal shifts upon incubation with *Bs*NusG-CTD (Fig. S6). Together, these data revealed an unexpected, albeit weak-affinity interaction between NusB and NusG, which involves the same portion of *Bs*NusB that interacts with *Bs*S10.

### *B. subtilis* NusG associates with S10

**Fig 6:**
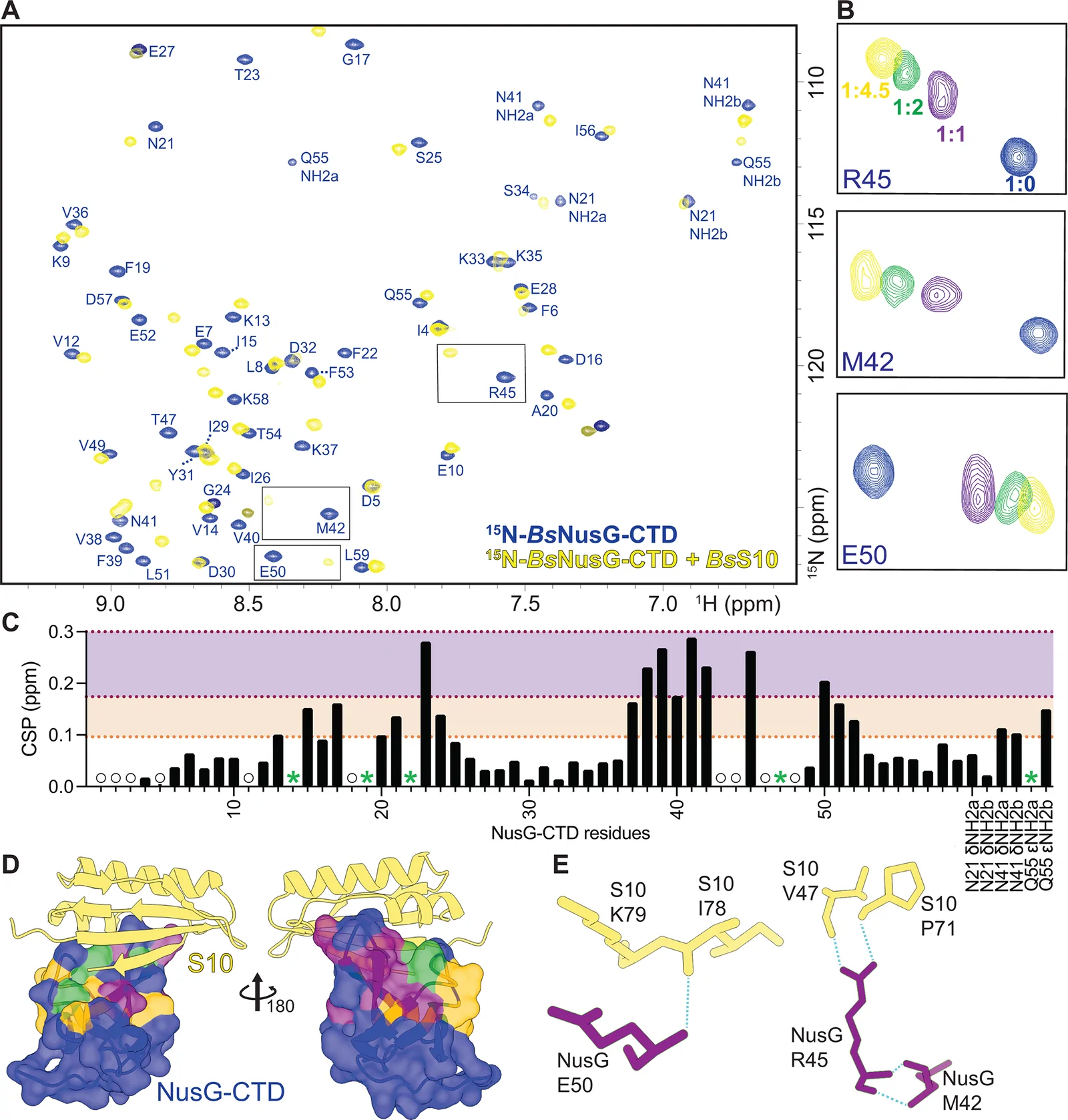
*B. subtilis* NusG associates with S10. (**A**) Overlay of ^1^H-^15^N HMQC spectra of ^15^N-labeled *Bs*NusG-CTD free in solution at 200 μM (blue) and in the presence of 4.48 molar equivalents of unlabeled S10 (yellow). (**B**) Unlabeled S10 was titrated into ^15^N-labeled *Bs*NusG-CTD with a molar ratio of 1.12× (purple), 2.24× (green), and 4.48× (yellow). Selected *Bs*NusG-CTD peaks that shifted significantly by more than 1 standard deviation above the average CSP are displayed. (**C**) Amide CSPs for *Bs*NusG-CTD determined as described in Fig. 1D. Green asterisks indicate residues that were assigned but whose corresponding peaks were strongly attenuated upon addition of S10. (**D**) AlphaFold 3 model of the *Bs*NusG-CTD:*Bs*S10 complex showing locations of *Bs*NusG-CTD residues with amide signal shifts either above the average CSP (orange), more than 1 standard deviation above the average CSP (purple), or exhibiting strong signal attenuation (green). (**E**) Representative residues from the NusG:S10 interface are shown with putative hydrogen-bonding interactions (blue dashed lines).

To test for an interaction between NusG and S10, we titrated ^15^N-labeled *Bs*NusG-CTD with unlabeled *Bs*S10 to a 4.5× molar ratio (Fig. 6A and B). ^1^H and ^15^N chemical shifts were analyzed for each titration point and plotted on a CSP graph (Fig. 6C). Most perturbed signals exhibited traceable shifts (fast exchange), while a handful of signals were completely attenuated after equimolar addition of *Bs*S10 (intermediate or slow exchange). We predicted the *Bs*NusG-CTD:*Bs*S10 complex by AlphaFold 3 and found that a subset of ^15^N-labeled *Bs*NusG-CTD residues with CSPs more than 1 standard deviation above the average are located within the predicted NusG:S10 interface, in agreement with the putative structural model (Fig. 6D). Among other interactions, several charged and significantly perturbed residues from *Bs*NusG-CTD, such as R45 and E50, are predicted to form electrostatic interactions with *Bs*S10 V74 and K79, respectively (Fig. 6E). Together, these data conclusively demonstrate that *B. subtilis* NusG forms a direct complex with S10, just as seen for the corresponding *E. coli* proteins.

### NusG:S10 complexes are also formed by other gram-positive species

**Fig 7:**
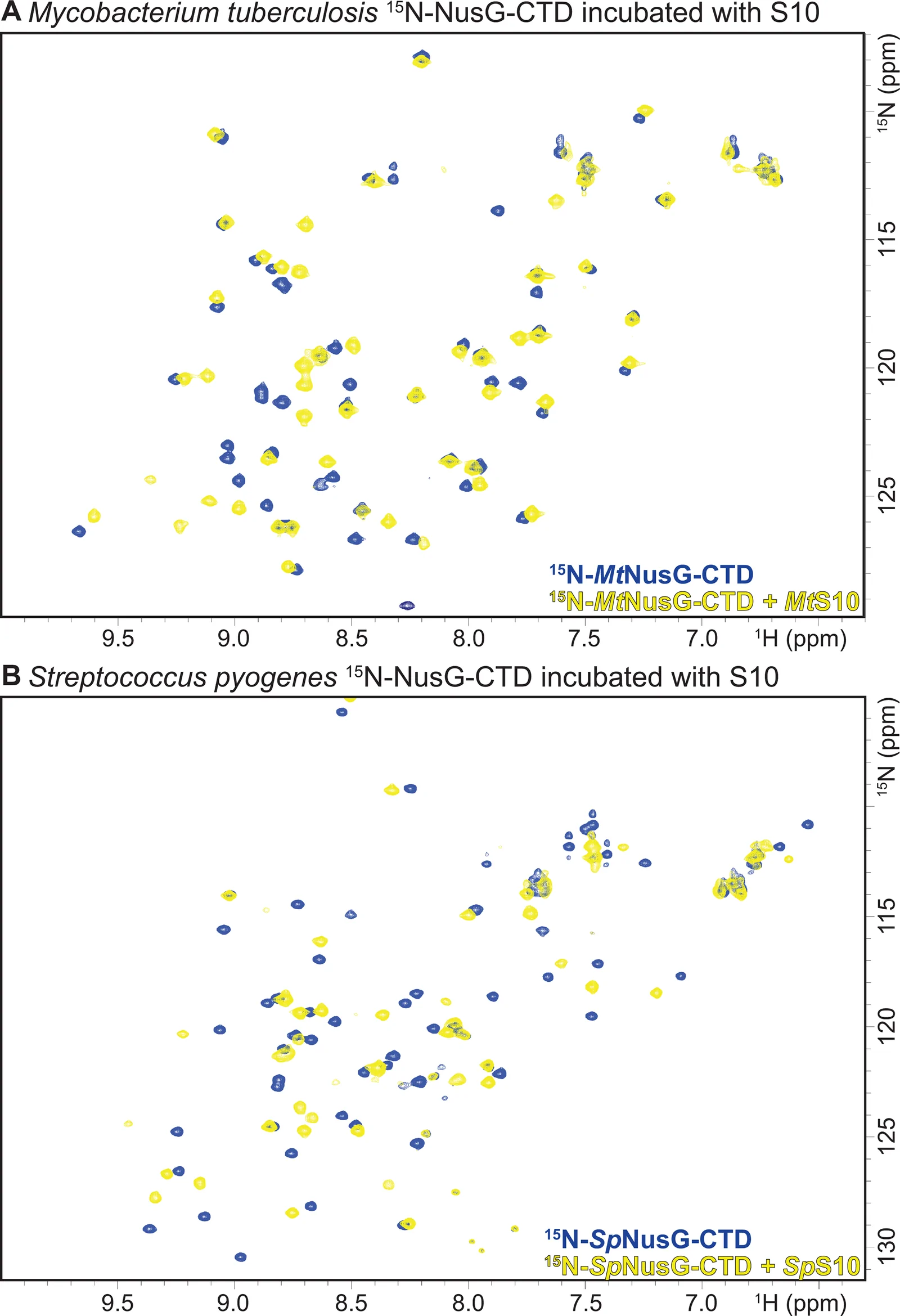
The NusG:S10 complex is formed by other gram-positive bacteria. (**A**) Overlay of ^1^H-^15^N HMQC spectra of ^15^N-labeled *M. tuberculosis* NusG-CTD free in solution at 200 μM (blue) and in the presence of two molar equivalents of unlabeled *M. tuberculosis* S10 (yellow). (**B**) Overlay of ^1^H-^15^N HMQC spectra of ^15^N-labeled *S. pyogenes* NusG-CTD free in solution at 200 μM (blue) and in the presence of two molar equivalents of unlabeled *S. pyogenes* S10 (yellow).

The NusG:S10 complex that has been demonstrated for *E. coli* is widely assumed to be formed by all related proteobacterial species. Given the interaction between *Bs*NusG and *Bs*S10 that we discovered herein, we wanted to examine whether similar complexes are also formed by other gram-positive species. To that end, we expressed and purified (unlabeled) S10 and ^15^N-labeled NusG-CTD from *Mycobacterium tuberculosis* and *Streptococcus pyogenes*. The S10 protein from each species was titrated into the cognate ^15^N-labeled NusG-CTD to a 2× molar ratio. In both cases, we observed clear residue-specific signal perturbations in the NusG-CTD spectra caused by the addition of S10 (Fig. 7). A majority of the perturbed signals from both *M. tuberculosis* and *S. pyogenes* NusG-CTD exhibited a fast-exchange binding regime, while a handful of signals from each species also showed a slow-exchange behavior. These observations closely resemble our NMR data for the *Bs*NusG-CTD:*Bs*S10 interaction. Therefore, our aggregate data demonstrate that NusG and S10 proteins are likely to form a protein:protein complex in gram-positive bacteria, even though these microorganisms are not thought to couple transcription and translation.

### *B. subtilis* NusG associates with Rho

**Fig 8:**
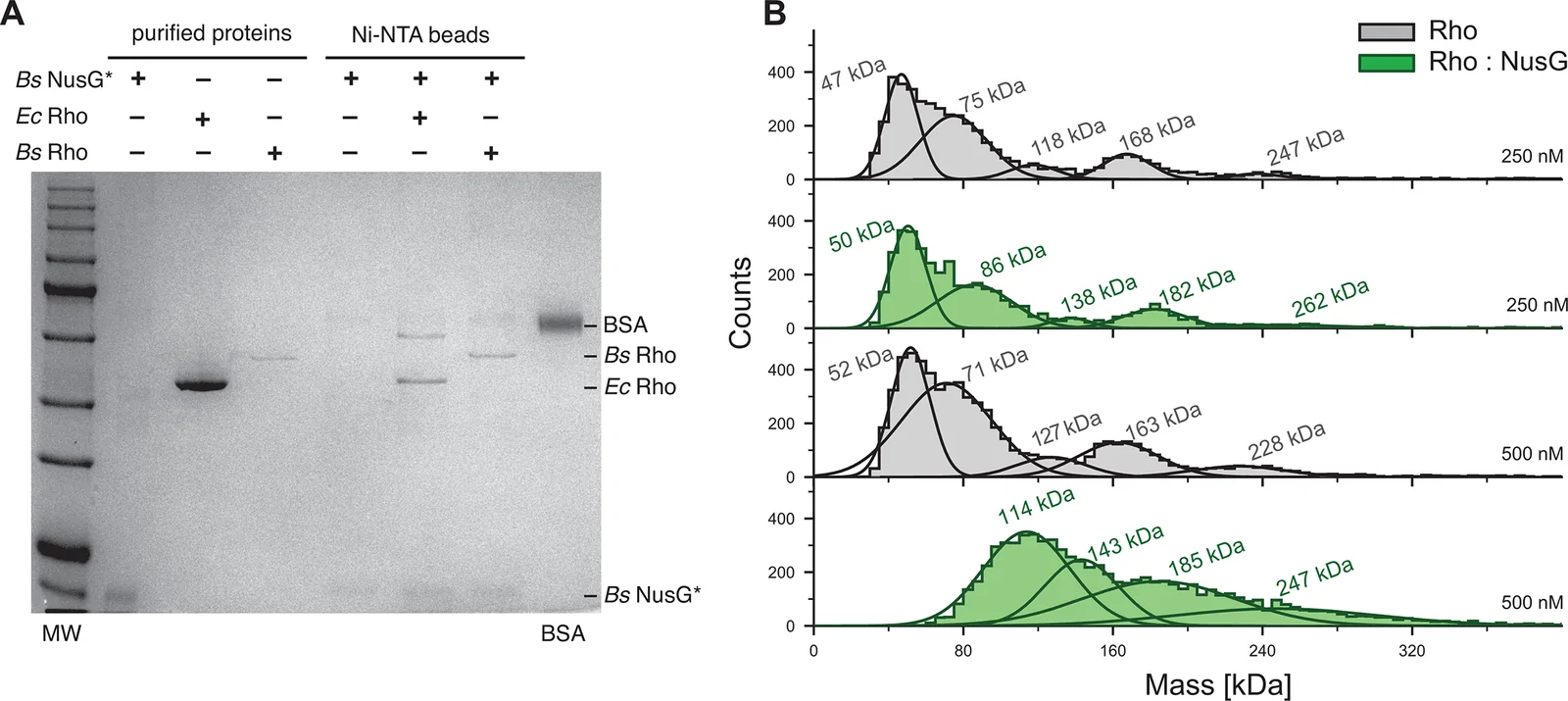
*B. subtilis* NusG associates with Rho. (**A**) Coomassie-stained gel shows the results of protein pull-down experiments. Purified proteins were loaded in the left lanes alongside a molecular weight marker (MW). Pull-down assays using Ni-NTA beads demonstrate that His-tagged *Bs*NusG copurifies with both *B. subtilis* Rho and *E. coli* Rho. * indicates an N-terminal His tag. A faint band observed in the pull-down reactions was confirmed to be BSA. (**B**) Mass photometry analysis of *Bs*Rho alone (gray) and *Bs*Rho with *Bs*NusG (green) at 250 nM and 500 nM reveals that *Bs*Rho populates multiple oligomeric species. The Rho oligomers associate transiently and dynamically, resulting in exchanging assemblies instead of fixed discrete states. Upon the addition of *Bs*NusG, *Bs*Rho higher-order oligomeric species show mass shifts at 250 nM that are further amplified at 500 nM, confirming Rho:NusG complex formation.

The *E. coli* NusG:Rho interaction plays a critical role in global transcription termination (20, 30). Given that Rho is dispensable in *B. subtilis*, it is unclear whether the *E. coli* model for transcription termination extends to gram-positive bacteria. The *Ec*Rho hexamer was previously reported to associate with monomeric NusG at a *K_d_* of 15 nM (25). However, structural studies have also suggested that the *Ec*Rho hexamer might associate with more than one *Ec*NusG-CTD (28, 29). In this study, we investigated whether *Bs*NusG could bind directly to *Bs*Rho *in vitro*, using purified proteins. Specifically, we employed pull-down experiments, wherein an affinity-tagged version of *Bs*NusG was found to interact with *Bs*Rho, as well as *Ec*Rho (Fig. 8A). We also employed mass photometry to investigate Rho:NusG interactions (Fig. 8B). Under our experimental conditions, *Bs*Rho existed predominantly in a monomeric or dimeric state until the concentration surpassed 750 nM, at which point dimers, tetramers, and hexamers became resolvable (Fig. 8B; Fig. S10). Unlike *Ec*Rho, which exhibits discrete oligomerization states when analyzed by mass photometry (53), our data show *Bs*Rho exchanging assemblies among different oligomeric states, suggesting weak self-assembly. This phenomenon has previously been observed by mass photometry for other proteins, which exhibited dynamic composition of multiple states (54, 55). The weak assembly of the *Bs*Rho hexamer, as compared to *Ec*Rho, has also been predicted by prior biochemical experimentation (56). Yet, despite this complex heterogeneity of *Bs*Rho, we observed clear mass shifts of *Bs*Rho oligomeric species upon the addition of BsNusG at concentrations of 500 nM or higher (Fig. 8; Fig. S10). Together, these data demonstrate an interaction between *Bs*NusG and lower oligomeric states of *Bs*Rho.

### Nus factor complexes are widely conserved in bacteria

**Fig 9:**
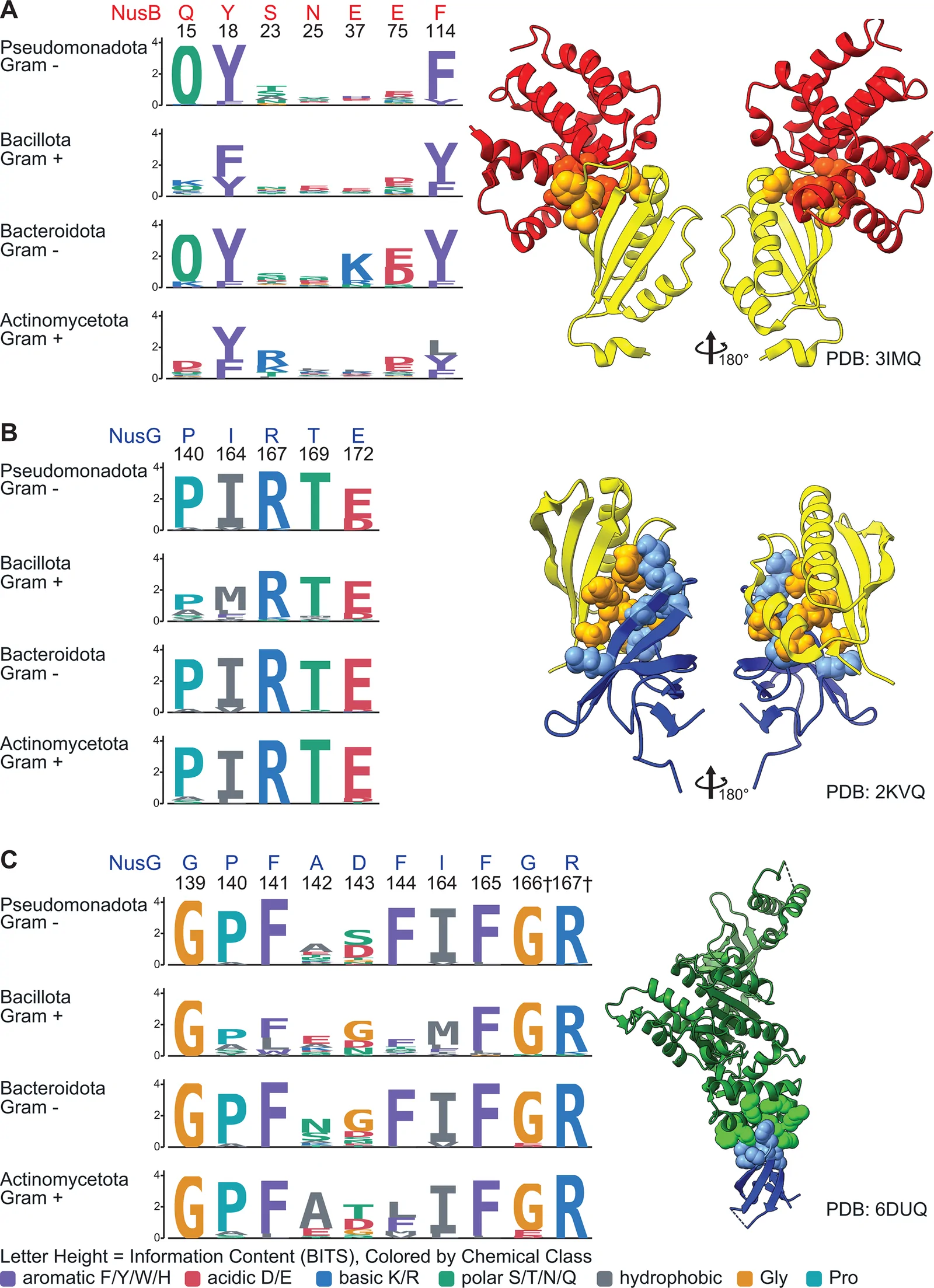
The residues involved in Nus factor complexes are broadly conserved in bacteria. (**A**) Sequence logos show the conservation of interface residues across four major bacterial phyla, Pseudomonadota (gram−), Bacillota (gram+), Bacteroidota (gram−), and Actinomycetota (gram+), for the NusB:S10 interface. *E. coli* residue positions are indicated above each column; letter height is proportional to information content (bits), and letters are colored by chemical class (aromatic F/Y/W/H, acidic D/E, basic K/R, polar S/T/N/Q, hydrophobic, Gly, and Pro). The corresponding interface is mapped onto the *E. coli* complex structure on the right (NusB, red; S10, yellow), shown in two orientations related by a 180° rotation. (**B**) As in panel **A** for the NusG:S10 interface (S10, yellow; NusG C-terminal domain, blue). (**C**) As in panel **A** for the NusG:Rho interface (Rho, green; NusG, blue).

Our overall data indicate that *B. subtilis* Nus factors associate together through a network of interactions that closely resembles that of *E. coli*. This raises the possibility that the Nus factor interaction network is broadly maintained across diverse bacterial lineages. To investigate this hypothesis, we acquired a large data set of NusB, S10, NusG, and Rho sequences from four major phyla (Pseudomonadota, Bacillota, Bacteroidota, and Actinomycetota). We then cataloged the key residues involved in the protein:protein interaction interfaces of *E. coli* NusB:S10, NusG:S10, and NusG:Rho and quantitatively examined the conservation of these contact residues (Fig. 9). This analysis revealed partial conservation in gram-positive bacteria for residues involved in the NusB:S10 complex. Y18 is a universally conserved aromatic residue, and F114 is an aromatic residue in most groups; however, Q15 is conserved in gram-negative bacteria but is replaced in gram-positive species. Also, some residues (S23/N25/E37) show modest variation in a lineage-dependent manner. Therefore, the NusB:S10 complex is likely to be widely utilized by bacteria, although with lineage-specific variation in the interface residues. By contrast, the NusG:S10 interface is highly conserved across diverse bacterial species. R167, T169, and E172 are essentially invariant in every phylum, including gram-positive bacteria. Similarly, P140 and I164 are strongly conserved. Finally, a set of core NusG:Rho interface residues (G139, F141, and F165) are also widely conserved, although this complex shows some variability within the A142-D143-F144 loop. Together, these data indicate that the NusB:S10, NusG:S10, and NusG:Rho complexes are likely to be universally found in bacteria, regardless of whether those organisms couple transcription and translation or exhibit runaway transcription in which the TEC outpaces the pioneering ribosome, as in *B. subtilis* (10).

## DISCUSSION

While much is known about gene expression in *E. coli*, the mechanisms often differ for *B. subtilis* and many gram-positive bacteria. For instance, *E. coli* Rho is essential and plays an important role in Rho-dependent termination across the genome, whereas *B. subtilis* Rho is not essential and only plays a minor role in termination (45, 46). Additionally, *Ec*NusG and *Ec*NusA are both essential in *E. coli*, whereas only NusA is essential in *B. subtilis*, suggesting fundamental differences in the molecular mechanisms used by the elongation factors (45). Also, *Bs*NusG functions as a sequence-specific RNA polymerase pausing factor. *Bs*NusG interacts with the nontemplate DNA strand within the transcription bubble at numerous regulatory pause sites (57–60), unlike *Ec*NusG, which functions as an anti-pausing factor without directly recognizing nontemplate DNA (61, 62). Furthermore, *Bs*NusG was discovered to promote termination at many intrinsic terminator sites (63, 64), whereas the relationship between *Ec*NusG and termination has not yet been resolved. Yet another critical difference between *B. subtilis* and *E. coli* is in their transcription and translation rates. When examined for a few reporter genes, the overall transcription and translation rates were found to be matched in *E. coli*. By contrast, *B. subtilis* transcription was shown to be nearly twice as fast as translation across those same genes (10, 44). These data suggest that transcription and translation remain uncoupled in *B. subtilis*. Based on this observation, it is possible that bacteria can be categorized into two groups: those that couple transcription and translation, like *E. coli*, and those that are not believed to engage in widespread transcription-translation coupling, such as *B. subtilis*. Although these categories do not align perfectly with “gram-negative” and “gram-positive” definitions, there is a strong overall trend supporting this classification. This fundamental difference in gene expression leaves a significant gap in our understanding of the regulation of transcription elongation in gram-positive bacteria, including the interaction network of elongation factors and the function and molecular composition of antitermination complexes (ATCs).

The interaction network of *E. coli* elongation factors can be thought of as falling into two categories. One network includes *boxA* RNA:NusB:S10:NusG, which, according to our bioinformatic data, is broadly conserved across gram-negative and gram-positive bacteria. This network of interactions is specifically required by ATCs, such as the *rrn* ATC. The second category includes the NusG:S10 interaction that drives transcription-translation coupling, along with the mutually exclusive NusG:Rho interaction that enhances termination. In this study, we directly investigated the corresponding *B. subtilis* proteins, starting with the interactions formed by NusB. The only reported function of *Ec*NusB is to associate with *boxA* RNA and S10 as part of an ATC that functions to block interference by Rho. Indeed, our data herein show that *Bs*NusB both associates with a *boxA*-like RNA sequence and with *Bs*S10. Therefore, by extension, our data suggest that ATCs are likely to be formed at *B. subtilis rrn* operons, and that these ATCs may closely resemble their *E. coli rrn* ATC counterparts. However, given that *B. subtilis* Rho is dispensable, additional experimentation is needed to determine whether the goal of the *B. subtilis rrn* ATC is exclusively to block Rho-dependent termination or if it is utilized for another purpose.

As part of our studies, we also discovered an unexpected interaction between *Bs*NusB and the *Bs*NusG-CTD. The regions of NusB and NusG that associate with S10 are also the portions that appear to participate in the NusB:NusG complex, indicating that NusG:S10 and NusB:S10 complexes are mutually exclusive with the NusB:NusG complex. However, it is unclear what role this protein:protein complex might serve and whether this observation is biologically meaningful, especially given the low binding affinity exhibited by the complex.

The *E. coli* NusG:S10 complex is an essential feature of the coupled transcription-translation machinery. However, transcription-translation coupling may not occur in *B. subtilis* due to a transcription rate that exceeds the translation rate (10, 44). Therefore, the *B. subtilis* NusG and S10 proteins might not be expected to form a complex if they are not needed for *B. subtilis rrn* expression and if they are not used as an intermachine bridge during transcription-translation coupling. Yet, our data herein conclusively demonstrate that a complex is formed between NusG and S10 in *B. subtilis*, *M. tuberculosis*, and *S. pyogenes*. Moreover, our bioinformatic analyses demonstrate that the NusG:S10 complex has been evolutionarily maintained in diverse bacteria. One potential explanation for this observation is that the NusG:S10 complex might have been evolutionarily retained solely to serve as a key feature of *rrn* ATC. Indeed, structural and biochemical analyses of the *E. coli rrn* ATC demonstrated that S10 is an important part of the extended exit channel that promotes rRNA folding and assists the initial steps of rRNA maturation (42). Thus, the NusG:S10 interaction is crucial for initially recruiting S10 to the TEC and further stabilizing the *rrn* ATC. Our discovery of a NusG:S10 interaction may also indicate that coupling transcription and translation is technically feasible, or perhaps transiently formed, in these bacteria. Recent research in *E. coli* showed evidence that transcription and translation coupling appears to be stochastic and not universal, and that the phenomenon heavily relies on the kinetics of the transcription machinery (65) and the efficiency of translation (66). Perhaps the kinetics of local transcription processivity can be altered such that transient coupling interactions are permitted. Alternatively, perhaps *B. subtilis* transcription and translation can be “loosely” coupled, akin to a recently observed structural intermediate of *E. coli* transcription-translation coupling (67). Such coupling would likely compete with the ability of *B. subtilis* NusG to interact with Rho in Rho-dependent termination, which is known to occur in *E. coli*. Therefore, while our data demonstrate that *E. coli* and *B. subtilis* share a common, and potentially universal, core network of elongation factor interactions, the function of some of these interactions is still unresolved in gram-positive bacteria.

## MATERIALS AND METHODS

### NMR spectroscopy

NMR experiments were performed on Bruker Avance III 600 MHz and 800 MHz spectrometers equipped with cryoprobes. All NMR samples were prepared in 25 mM Tris buffer at pH 8.0 with 100 mM NaCl, 1 mM TCEP, 5%–10% D_2_0 and analyzed at 25°C. Samples of ^15^N-labeled proteins used for titration experiments were prepared at an initial concentration of 0.2–0.25 mM, and 2D ^1^H-^15^N SOFAST-HSQC spectra were recorded at each titration point. NMR data were processed with TopSpin 4.1.4 (Bruker Biospin) and analyzed with Sparky (68). Amide chemical shift perturbations (CSPs) were calculated for each signal/residue using the following equation (69):


(1) $$\Delta \delta =\;\sqrt{{\left(\Delta \delta_{H}\right)}^{2}+{\left(\Delta \delta_{N}/5\right)}^{2}}$$

where $\Delta \delta_{H}$ and $\Delta \delta_{N}$ are chemical shift perturbations for the ^1^H and ^15^N resonances, respectively.

The dissociation constant, *K*_*d*_, was calculated by fitting experimental CSPs at respective titration points to a single-site binding model using an in-house MATLAB program Kdfit (52). The CSPs observed during titration were analyzed using the following equation:


(2) $$\Delta \delta =\Delta \delta_{max}\frac{\left[P_{t}\right]+\left[L_{t}\right]+K_{d}-\sqrt{{\left(\left[P_{t}\right]+\left[L_{t}\right]+\left[K_{d}\right]\right)}^{2}-4\left[P_{t}\right]\left[L_{t}\right]}}{2\left[P_{t}\right]}$$

where $\;\Delta \delta_{max}$ is the CSP value at complete saturation, $\left[P_{t}\right]$ is the total protein concentration, $\left[L_{t}\right]$ is the total ligand concentration, and $K_{d}$ was treated as the global fitting parameter.

Samples of ^15^N/^13^C-labeled NusB and ^15^N-labeled NusG-CTD used for backbone and side-chain resonance assignment were prepared at 0.2–0.5 mM concentration. Standard Bruker double- and triple-resonance experiments performed for NusB and NusG-CTD signal assignment included 2D ^1^H-^15^N SOFAST-HSQC and 3D ^15^N-edited TOCSY and NOESY; 3D HNCACB, H(CC)(CO)NH, and (H)CC(CO)NH.

### Pull-down experiments with His-tagged proteins

N-terminal His-tagged *B. subtilis* NusG, *B. subtilis* Rho, and *E. coli* Rho were purified as described previously (57, 70). Three aliquots of 100 μL of Ni-NTA agarose beads (Qiagen) were washed three times with 200 μL of interaction buffer (20 mM Tris-HCl, pH 7.9, 100 mM KCl, 1 mM DTT, 0.1% Triton X-100, and 20 mM PMSF). The beads were then mixed with 50 pmol of His-tagged NusG in 500 μL of interaction buffer. The tubes were then incubated at 4°C for 2 h in a shaking incubator. The beads were washed three times with 500 μL of low adsorption buffer (interaction buffer supplemented with 0.1% BSA and 20 mM imidazole). Next, 150 pmol of untagged Rho protein (*B. subtilis* or *E. coli*) in 500 μL of low adsorption buffer was added and then incubated at 4°C for 16 h in a shaking incubator. A volume of 500 μL of low adsorption buffer was added to the control tube without added Rho. Samples were then centrifuged, and the supernatant was discarded. The beads were washed with 800 μL of low adsorption buffer, pelleted, and then resuspended in gel loading buffer (2% SDS, 12% glycerol, 0.1 mM Tris-HCl, pH 6.8, 0.35 M 2-mercaptoethanol, 0.01% bromophenol blue, and 100 mM imidazole). Samples were heated at 90°C for 5 min. Samples were electrophoresed through a 15% SDS-PAGE gel together with purified proteins and a protein size marker (GeneTex). Gels were stained with Coomassie blue.

### Isothermal titration calorimetry

ITC data were collected using the Affinity ITC instrument. Prior to experiments, all proteins were dialyzed in the same beaker containing 2 L of ITC buffer with 25 mM Tris-HCl, pH 8, 100 mM NaCl, and 1 mM DTT overnight before the ITC experiment. *Bs*S10 (700 μM) was titrated into either *Bs*NusB (70 μM) or the buffer alone for blank measurements. The experiments were performed at 25°C, with a stirring rate of 125 rpm, and an initial cell volume of 182 μL, using a 0.5 μL first injection, followed by 20 injections of 3 μL each. The blank measurements were subtracted from the experimental data to account for the heat of dilution. Data were analyzed by NanoAnalyze and GraphPad Prism 10.

### Mass photometry

Mass photometry measurements of purified Rho and NusG were collected on a Refeyn TwoMP instrument using AcquireMP software. Proteins were diluted to a working concentration of 10 μM in 25 mM Tris-HCl, pH 8, 300 mM NaCl buffer. Proteins were then incubated together at 1 μM at room temperature for 15 min before being further diluted to various concentrations for each 20 μL droplet reading. Mass calibrations were performed using 1× MP1 calibrants from Refeyn and 20 nM BSA. Data analysis was performed using DiscoverMP software by Refeyn. Data from the video recordings were plotted as histograms (bin width 5) of ratiometric contrast to scattering signals, and mass standard calibration was applied to all readings to identify Rho oligomers and mass shifts.

### Bioinformatic analysis of interface conservation

Interface-residue conservation was assessed from NusB and NusG protein sets derived from GTDB release R226 (71) representative genomes. Canonical NusG genes (*n* = 114,633) were resolved from their paralogs (RfaH, LoaP, ActX, UpxY, and TaA) and archaeal Spt5 by phylogenetic clade-typing of the COG0250 family, and NusB genes (*n* = 127,906) were taken as COG0781 members after removing RsmB methyltransferase (COG0144) and PHP-domain contaminants. Genes were assigned by GTDB taxonomy to the four most populated phyla (Pseudomonadota, Bacillota, Bacteroidota, and Actinomycetota). Within each phylum, sequences were dereplicated with CD-HIT (70% identity) (72), aligned to the *E. coli* reference (NusB, P0A780; NusG, P0AFG0) with MUSCLE5 (73), and the amino acid frequency at each interface position was tabulated and rendered as sequence logos (letter height = information content in bits, colored by chemical class).

## Data Availability

All primary data are deposited on an archived site on the Mendeley Data website and can be found at “Tran Peterson et al Interaction Network of *B. subtilis* Elongation Factors," Mendeley Data, V1, doi: 10.17632/sjnkwyjw68.1. The NMR signal assignments for *Bs*NusB and *Bs*NusG-CTD have been deposited in the Biological Magnetic Resonance Data Bank (BMRB) under the accession numbers 54026 and 54027, respectively.

## SUPPLEMENTAL MATERIAL

Supplemental Figures mbio.02180-26-s0001.pdf Figures S1 to S10.

ASM does not own the copyrights to Supplemental Material that may be linked to, or accessed through, an article. The authors have granted ASM a non-exclusive, world-wide license to publish the Supplemental Material files. Please contact the corresponding author directly for reuse.
