# Supplementary material for: The core interaction network of transcription elongation factors is broadly maintained across diverse bacteria
Source: mBio. 2026 Oct 23;17(11):e02180-26. doi: 10.1128/mbio.02180-26 (PMC13753749; doi:10.1128/mbio.02180-26)
Supplement: Supplementary file 1 [file mbio.02180-26-s0001.pdf]

Supplementary Materials

Supplementary Figures

Figure S1: Alignment of *B. subtilis rrn* leader regions reveals putative *boxA* sequences
Figure S2: SDS-PAGE of purified elongation factors
Figure S3: CD of *Bs*NusB and *Bs*NusB^{Q20A,S20L} and ITC data for *Bs*S10:*Bs*NusB^{Q20A,S20L}
Figure S4: NMR assignments for *Bs*NusB
Figure S5: Analysis of the *Bs*NusB:*boxA* complex
Figure S6: Venn diagram of ^{5}N-*Bs*NusB residues affected by interactions with binding partners studied here
Figure S7: NMR analysis of the *Bs*NusB:*Bs*NusG and *Bs*NusB:*Bs*NusG-CTD interactions
Figure S8: NMR analysis of ^{15}N-*Bs*NusB interactions with *Bs*NusG-NTD
Figure S9: NMR signal assignments for *Bs*NusG-CTD
Figure S10: Mass photometry analyses indicate that *Bs*NusG associates with *Bs*Rho

```
rrnO      GTCGCTTT---GAGAGAAGCACACAAAGTTCTTTGAAAACTAAACAAGACAAAACGTACC     57
rrnD      CGCTTCAA---CAAGAA--GTGAAAATGATCTTTGAAAACTAAACAAGACAAAACGTACC     173
rrnB      TGCTTCAT---TGAGAAGTAACGAAATGATCTTTGAAAACTAAACAAGACAAAACGTACC     343
rrnE      ----------------CATTATTGAATGATCTTTGAAAACTAAACAAGACAAAACGTACC     44
rrnA      CGCTTAAAC----GAGCGGTAAACAAAGTTCTTTGAAAACTAAACAAGACAAAACGTACC     190
rrnI      TGCTTCGT-----TAAGCGGCAGTAATGATCTTTGAAAACTAAACAAGACAAAACGTACC     296
rrnJ      CGCTTGAAC----GAAGTGAACAAAATGATCTTTGAAAACTAAACAAGACAAAACGTACC     255
rrnG      TGCTTTAACAAAGCGGACAAACAAAATGATCTTTGAAAACTAAACAAGACAAAACGTACC     129
rrnW      TGCTTTAACAAAGCGGACAAACAAAATGATCTTTGAAAACTAAACAAGACAAAACGTACC     117
rrnH      TGCTTTAACAAAGCGGACAAACAAAATGATCTTTGAAAACTAAACAAGACAAAACGTACC     117
                                  ** * *******************************

rrnO      TGTTAATTCATTTTT--ATAAATCGCACAGCGATGTGCGTAGTCAGTCAAACTAGGGCCT     115
rrnD      TGTTAATTCAGTTTTTAAAAAATCGCACAGCGATGTGCGTAGTCAGTCAAACTAGGGCCT     233
rrnB      TGTTAATTCAGTTTTT-AAAAATCGCACTGCGATGTGCGTAGTCAGTCAAACTAGGGCCT     402
rrnE      TGTTAATTCAGTTTTT-GAAAATCGCACAGCGATGTGCGTAGTCAGTCAAACTAGGGCCT     103
rrnA      TGTTAATTCATT-TTT-ATAAATCGCACAGCAATGTGCGTAGTCAGTCAAACTAC-----     243
rrnI      TGTTAATTCATT-TTT-ATAAATCGCACAGCGATGTGCGTAGTCAGTCAAACTAC-----     349
rrnJ      TGTTAATTCAGTTTTT-AAAAATCGCACAGCGATGTGCGTAGTCAGTCAAACTAC-----     309
rrnG      TGTTAATTCAGTTTTT-AAAAATCGCACAGCGATGTGCGTAGTCAGTCAAACTAC-----     183
rrnW      TGTTAATTCAGTTTTT-AAAAATCGCACAGCGATGTGCGTAGTCAGTCAAACTAC-----     171
rrnH      TGTTAATTCAGTTTTT-AAAAATCGCACAGCGATGTGCGTAGTCAGTCAAACTAC-----     171
          ********** * **    ********* ** **********************

rrnO      GCACGACGCAGGTCACACAGGTGTCGCCGCAGGATGCGGTGAACTTAACCTGTGATCC       173
rrnD      GCACGACGCAGGTCACACAGGTGTCGCCGCAGGATGCGGTGAACTTAACCTGTGATCC       291
rrnB      GCACGACGCAGGTCACACAGGTGTCGCCGCAGGATGCGGTGAACTTAACCTGTGATCC       460
rrnE      GCACGATGCAGGTCACACAGGTGTCACCGCGGGATGCGGTGGACTTAACCTGTGATCC       161
rrnA      ----------------------------------------------------------       243
rrnI      ----------------------------------------------------------       349
rrnJ      ----------------------------------------------------------       309
rrnG      ----------------------------------------------------------       183
rrnW      ----------------------------------------------------------       171
rrnH      ----------------------------------------------------------       171
```

Figure S1: Alignment of *B. subtilis rrn* leader regions reveals putative *boxA* sequences. *B. subtilis* encodes 9 or 10 separate *rrn* operons, depending on the strain. Shown in this figure is a portion of the region located just upstream of the 16S gene in each of the operons from the undomesticated strain NCIB 3610. Nine of the operons appear to potentially form a small hairpin (shown in red), which is located just upstream of a *boxA*-like sequence (shown in blue). The *rrnO boxA* sequence was investigated further in this study.

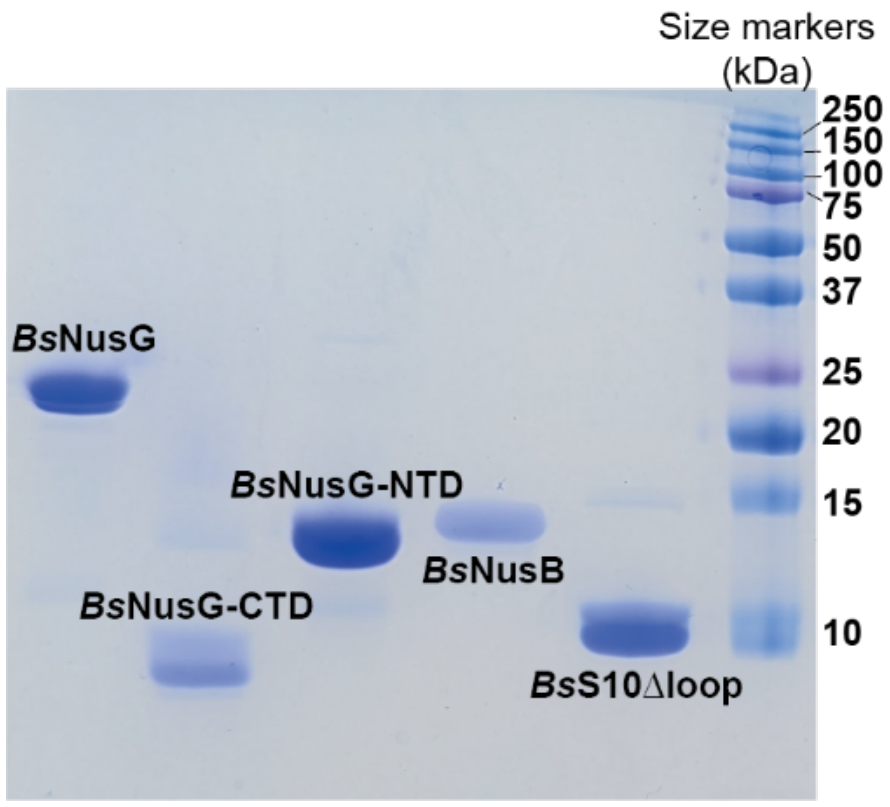

Figure S2: SDS-PAGE gel of purified elongation factors used in the study for NMR analyses. From the left to right, lanes contain *Bs*NusG, *Bs*NusG-CTD, *Bs*NusG-NTD, *Bs*NusB and *Bs*S10$^{\Delta\text{loop}}$

<u>Protein overexpression and purification</u>

All S10 constructs of *B. subtilis, M. tuberculosis* and *S. pyogenes* were made with the removal of intrinsically disordered loop position, corresponding to residues 46-67 for *Ec*S10. This portion was replaced in all instances by a serine residue. All proteins were expressed in *E. coli* T7 Express cells using N-terminal 10xHis-SUMO fusion constructs. Using 5 mL of an overnight starter culture, 1.5 L of 2xYT media were inoculated, incubated, shaking at 37 °C to an OD_{600} of 0.4 - 0.6, and then induced with 1 mM IPTG. The cultures were moved to room temperature and allowed to shake for an additional 18 h. Cells were then harvested by centrifugation at 5,000 g for 20 min.

Cell pellets (3 g) were resuspended in lysis buffer containing 50 mM Tris-HCl pH 8, 1 M NaCl, 1 mM DTT, 100 μM PMSF, and lysed by sonication (30% power, 60 output setting, 6 cycles of 12 s on and 48 s off). Polyethyleneimine (PEI) and ammonium sulfate fractionation were then performed (1) using a final concentration of 0.5% PEI and 20%-60% ammonium sulfate. Ammonium sulfate protein pellets were collected via centrifugation and resuspended in 30 mL of Ni-binding buffer containing 50 mM Tris-HCl, pH 8, 1 mM DTT, 25 mM imidazole, and with either 1 M NaCl (for S10) or 300 mM NaCl (for NusG, NusB). The ammonium precipitate resuspensions were incubated with Ni-NTA resin for at least 2 h. The column was then washed with 5 column volumes of binding buffer and 15 column volumes of wash buffer containing 50 mM imidazole. Proteins were eluted with 15 mL elution buffer containing 250 mM imidazole. bdSENp1 protease was added for removal of the His-SUMO tag, and eluates were then dialyzed against 50 mM Tris-HCl, pH 8, 300 mM NaCl or (1 M for S10), 1 mM DTT and 1 mM $MgCl_2$. Following overnight cleavage reactions, the cleaved samples were incubated with Ni-NTA resin for the removal of the cleaved tag and uncleaved protein. A final purification step was performed by Superdex 75 size-exclusion chromatography with the flow rate of 0.5 mL/min. Purified proteins were collected, concentrated by concentrator spin columns, and dialyzed into a storage buffer containing 25 mM Tris HCl, pH 8, 100 mM NaCl, and 1 mM DTT. Protein identity and their overall purity were confirmed using SDS-PAGE gels and by analysis on a JEOL AccuTOF ESI mass spectrometer.

A

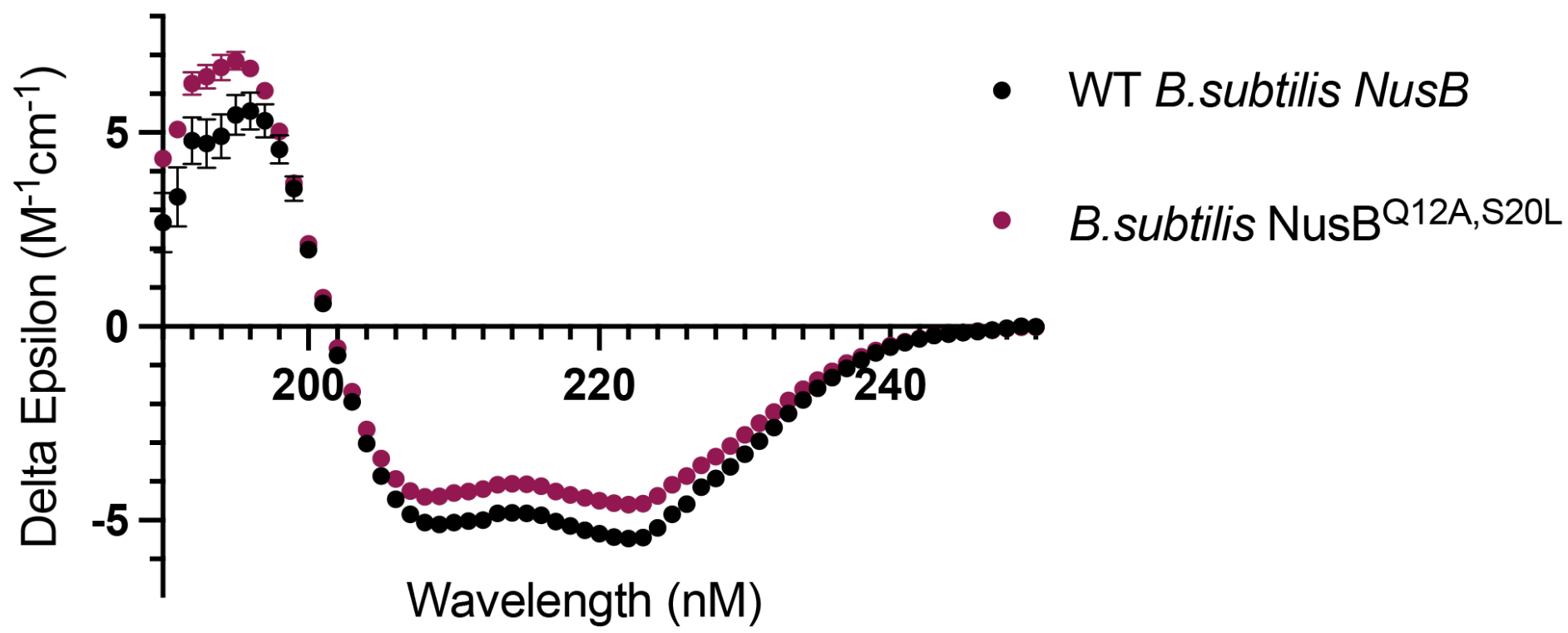

B

	Secondary structure content in %				
	Helix	Antiparallel	Parallel	Turn	Others
WT NusB	40.8	7.2	0.1	7.4	44.6
NusB $^{Q12A\ S20L}$	42.3	1.5	0.6	9.2	46.5

C

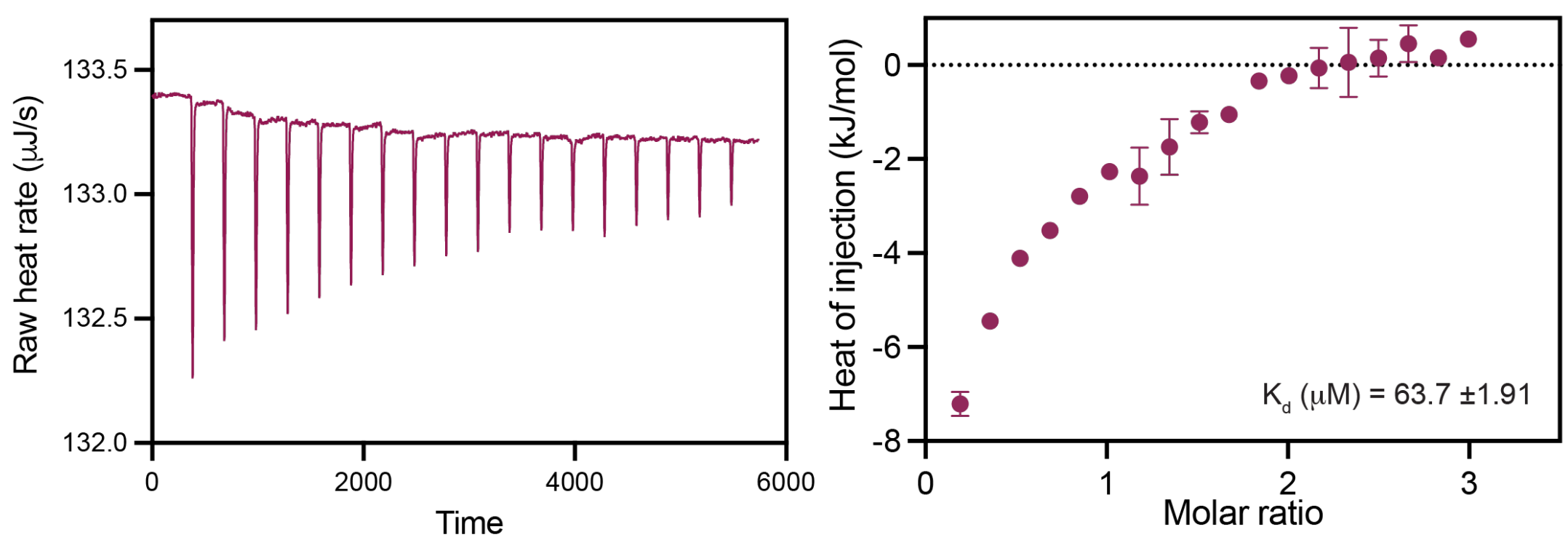

Figure S3: (A) Circular dichroism spectra of wild-type *Bs*NusB and *Bs*NusB^{Q12A,S20L}. All proteins were dialyzed against 2 L of 10 mM sodium phosphate, pH 7.4, and 100 mM NaF, and then diluted to a concentration of 0.2 mg/mL concentration. These 0.5 ml samples were transferred to a 1 mm quartz cuvette for readings. The experiments were performed in triplicate on Jasco J-810 circular dichroism spectrometer with a measuring range of 190 – 260 nm, 50 nm/min speed, 1 nm data pitch, 1 nm bandwidth, and for 3 accumulation scans. The data analysis was performed and collected by BestSel online data website. (B) Table data of BestSel analysis showing the percentage of each secondary structure element in both proteins. The similar secondary structure content of the two proteins indicates that the substitution of Q12A and S20L did not significantly alter the overall protein fold. (C) Isothermal titration calorimetry analysis of the interaction between *Bs*S10 versus *Bs*NusB^{Q20A,S20L}. Under the identical experimental conditions, the mutant protein exhibits reduced binding affinity for S10 compared to wild-type *Bs*NusB.

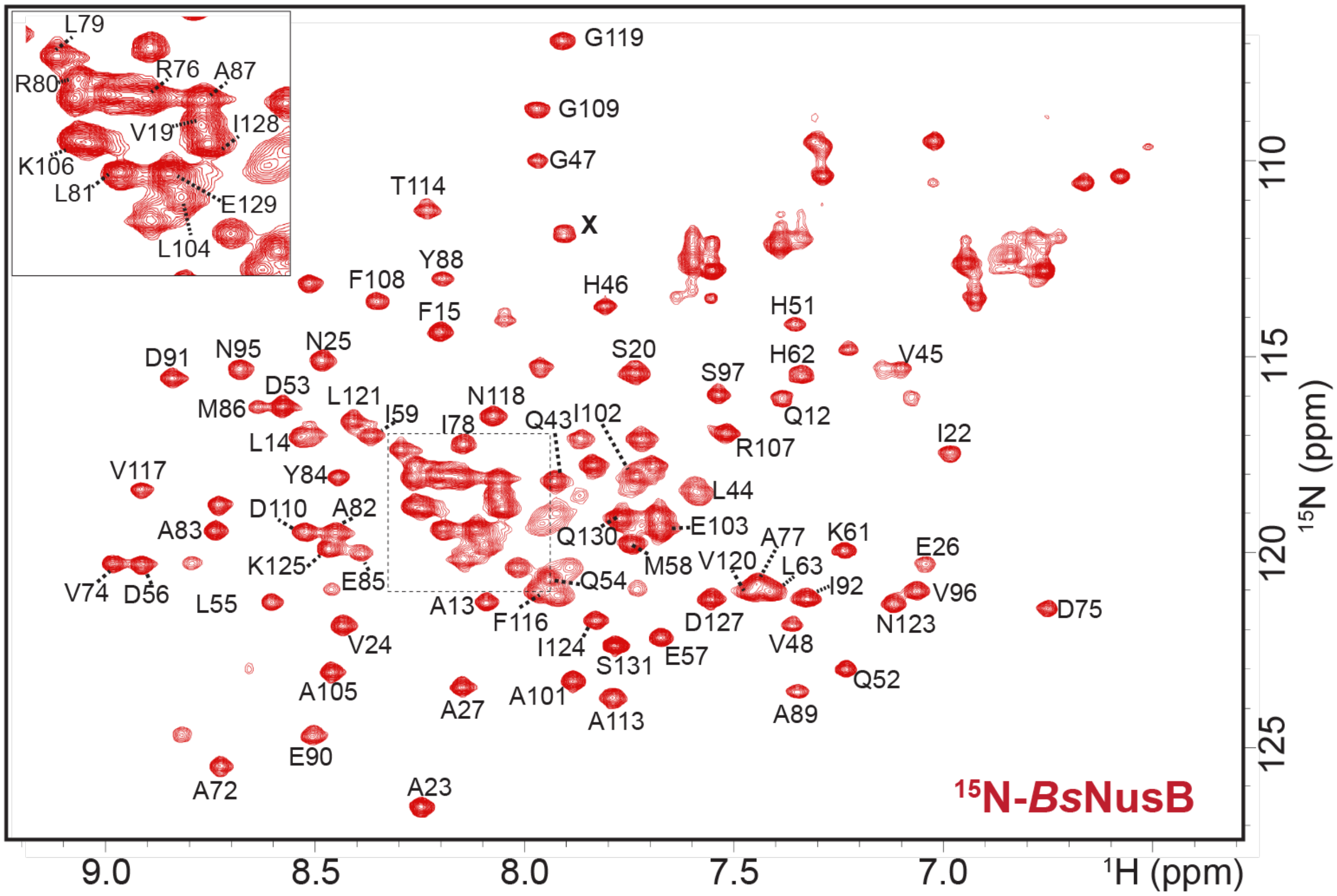

*Bs*NusB Sequence:
MKRRTAREKALQALFQIDVSDIAVNEAIEHALDEEKTDPFFEQLVHGVLEHQDQLDEMISKHLVNWKLDRIANVDRAIL RLAAYEMAYAEDIPVNVSMNEAIELAKRFGDDKATKFVNGVLSNIKSDIEQS

Figure S4: ^{1}H-^{15}N HMQC spectrum of 200 μM ^{15}N-labeled *Bs*NusB at 25°C. Backbone amide NMR signals assigned to specific residues are labeled with the corresponding residue type and number. Signal labeled X is unassigned but used to calculate K_d value in Figure S7C. Also shown is the amino acid sequence of *Bs*NusB.

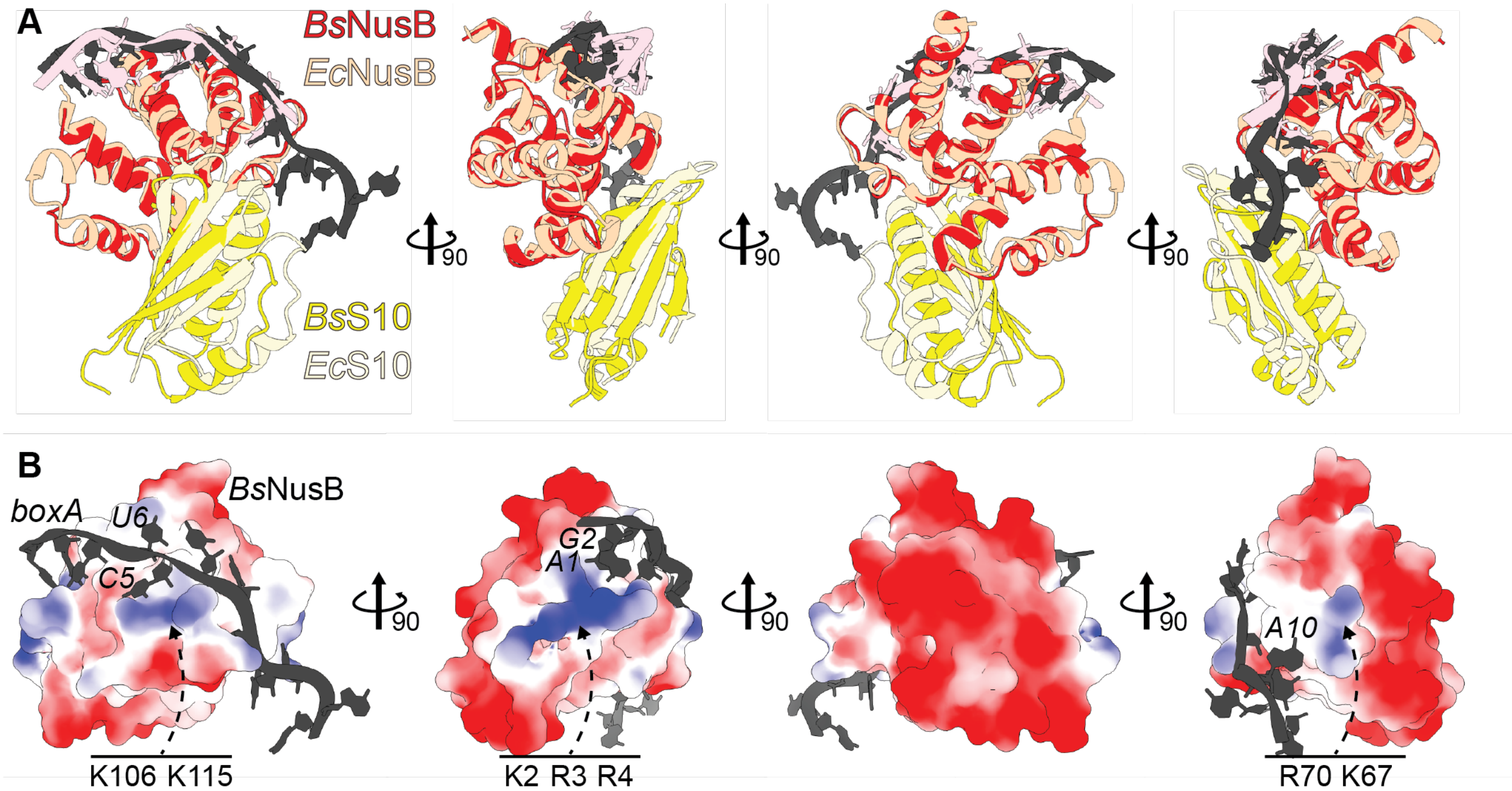

Figure S5: Analysis of the *Bs*NusB:*boxA* complex. (A) Comparison of *Ec*NusB:S10 complex (PDB: 3D3C) with the predicted structure of *Bs*NusB:S10. The *Bs*NusB:*boxA* complex appears to rely on electrostatic interactions and direct interactions between amino acid side chains and *boxA* nucleotides. Specifically, nonbridging oxygens from nucleotides U6 and G9 are likely to exhibit charge-charge interactions with *Bs*NusB residues K115 and K112, respectively. Nucleotides A1 and G2 are adjacent to a positive patch comprised of residues K2, R3, and R4, and A10 is close to R70. A few residues that are predicted to interact directly with *boxA* nucleotides are N95 and K125, which look to form interactions with U4. Additionally, residues E103 and K106 are likely to associate with *boxA* C5. Similarly, N123 is likely to associate with *boxA* U7. I92 may also form a stacking interaction with nucleotides A1 and G2. Finally, D91 is likely to contact R3, which may interact with *boxA* G2. The protein:protein interface of the *Bs*NusB:S10 and *Bs*S10:NusG complexes also generally resemble the *E. coli* counterparts. (B) AlphaFold3 model of the *Bs*NusB:*boxA* RNA complex showing an electrostatic surface potential map for NusB.

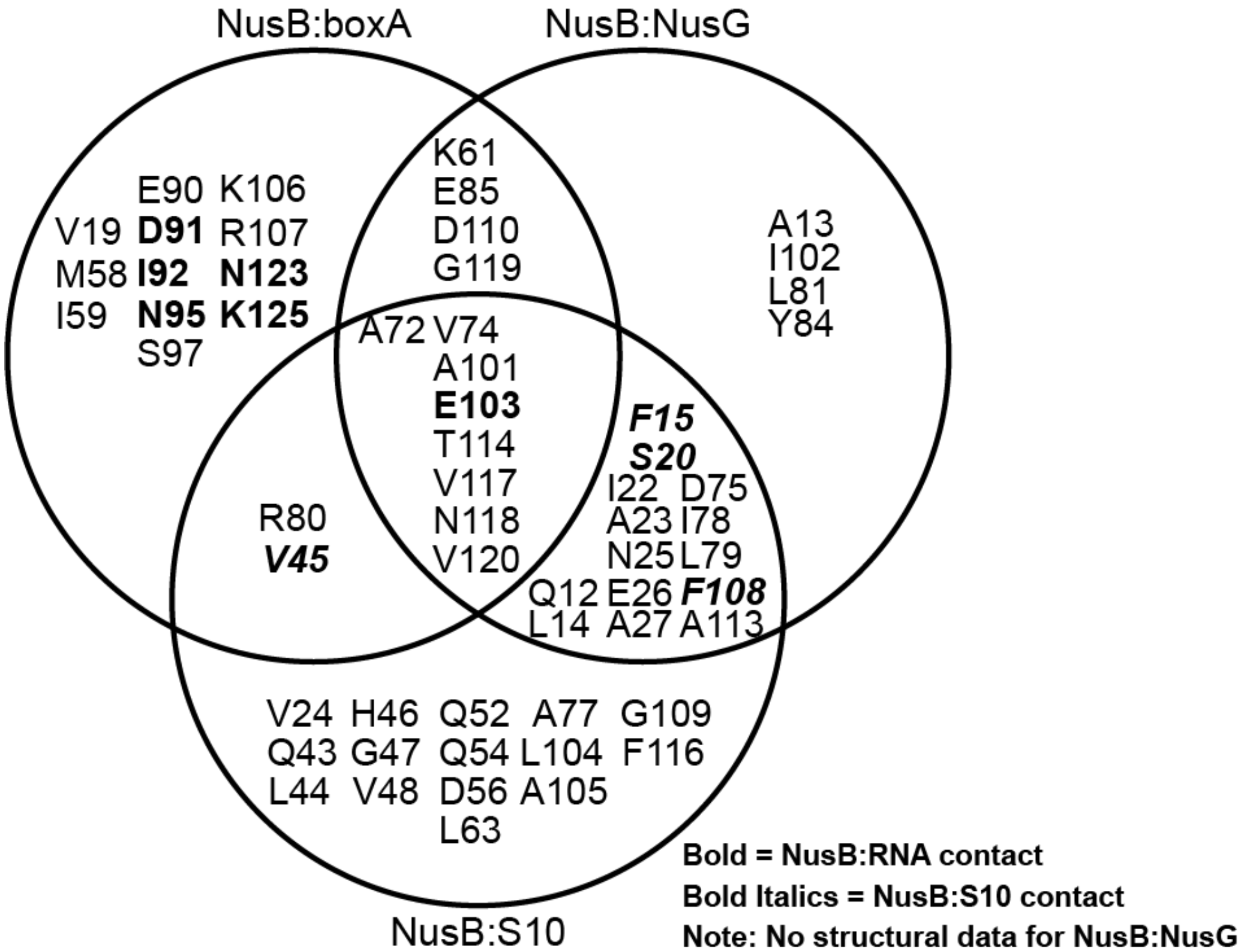

Figure S6: Venn diagram of ^{15}N-*Bs*NusB residues that exhibit significant CSP shifts within the *Bs*NusB:*boxA*, *Bs*NusB:*Bs*NusG, and *Bs*NusB:*Bs*S10 complexes.

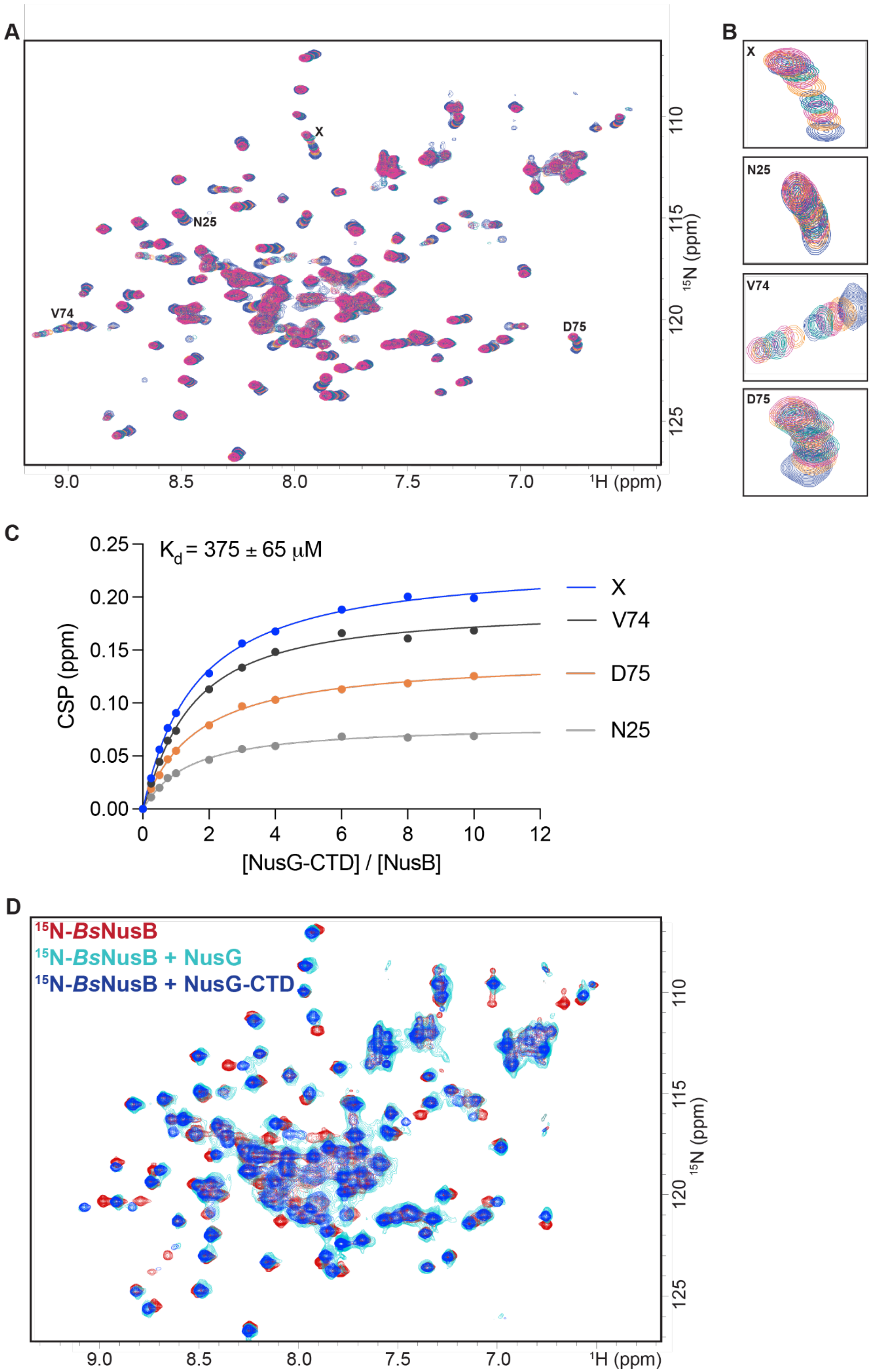
A
X
N25
V74
D75
9.0
8.5
8.0
7.5
7.0
^{1}H (ppm)
110
115
120
125
^{15}N (ppm)
B
X
N25
V74
D75
C
K_d = 375 ± 65 μM
CSP (ppm)
0.00
0.05
0.10
0.15
0.20
0.25
[NusG-CTD] / [NusB]
0
2
4
6
8
10
12
X
V74
D75
N25
D
^{15}N-*Bs*NusB
^{15}N-*Bs*NusB + NusG
^{15}N-*Bs*NusB + NusG-CTD
9.0
8.5
8.0
7.5
7.0
^{1}H (ppm)
110
115
120
125
^{15}N (ppm)

Figure S7: NMR analysis of *Bs*NusB:*Bs*NusG interactions. (A) Overlay of ^{1}H-^{15}N HMQC spectra of ^{15}N-labeled *Bs*NusB recorded at various points in the course of titration with increasing amounts of unlabeled *Bs*NusG-CTD, up to 10x molar ratio. (B) Zoom on select signals from the spectra shown in panel A. (C) Titration curves showing global fit of the CSPs for select residues (from panel B) to Equation (2) in the main text. The resulting K_D value was 375 μM. (D) Overlay of ^{1}H-^{15}N HMQC spectra of ^{15}N-labeled *Bs*NusB alone (red) and in the presence of 2 molar equivalents of unlabeled full-length *Bs*NusG (teal) and *Bs*NusG-CTD (blue).

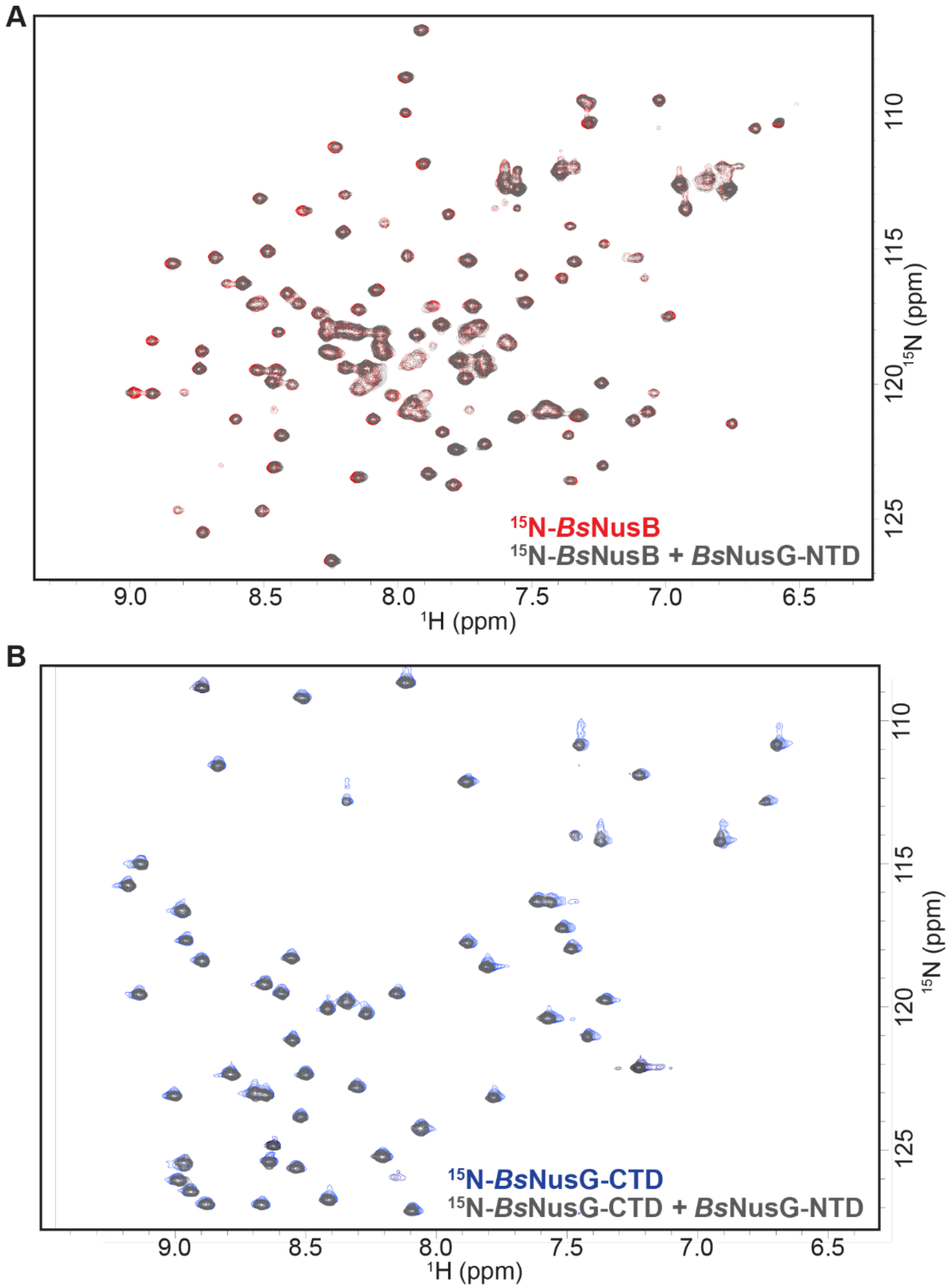

Figure S8: (A) Overlay of ^{1}H-^{15}N HMQC spectra of ^{15}N-labeled *Bs*NusB alone (red) and in the presence of 2X molar equivalent amount of unlabeled *Bs*NusG-NTD (gray). (B) Overlay of ^{1}H-^{15}N HMQC spectra of ^{15}N-labeled *Bs*NusG-CTD alone (blue) and in the presence of 2X molar equivalent amount of unlabeled *Bs*NusG-NTD (gray)

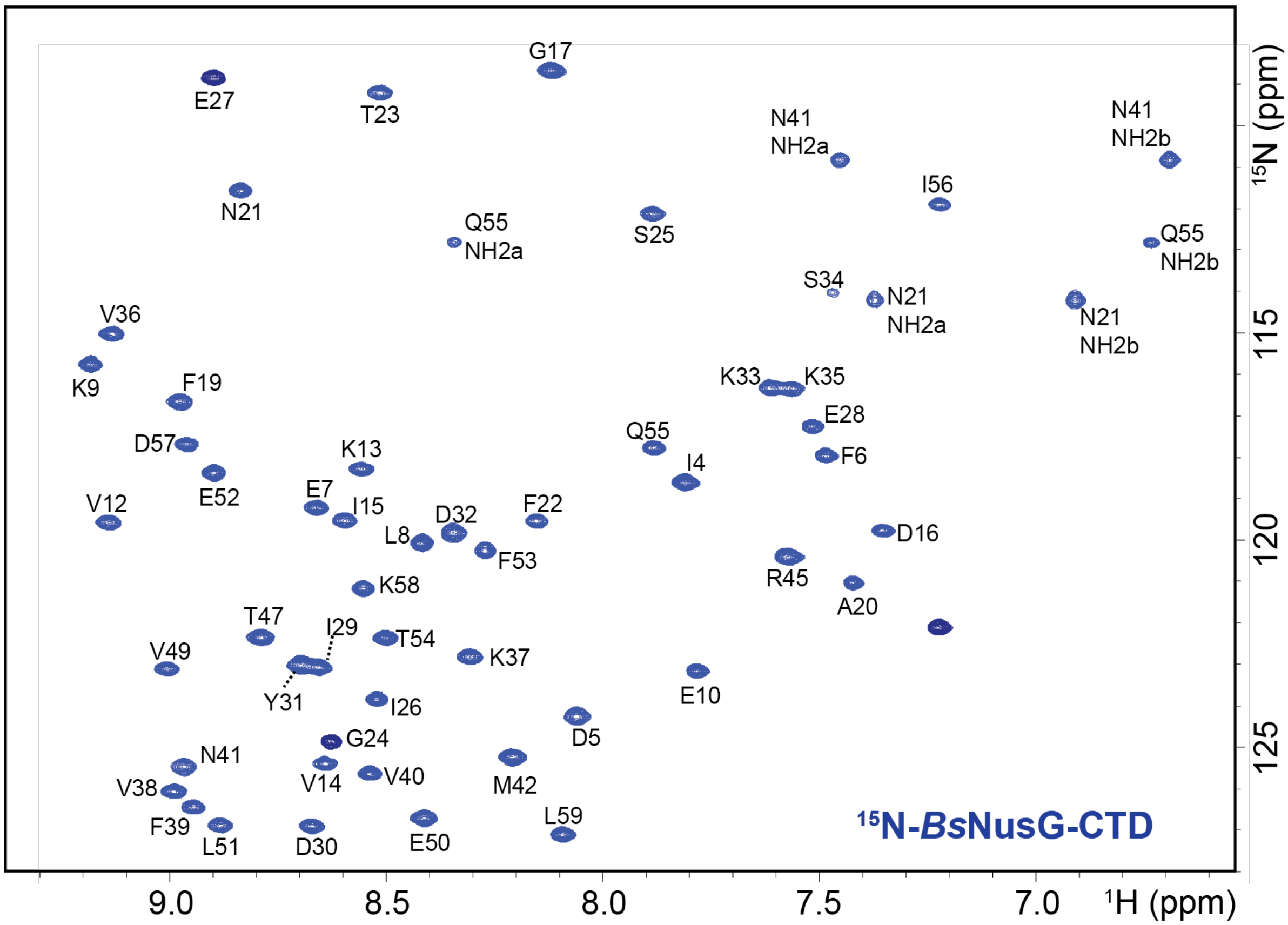

*Bs*NusG-CTD Sequence (peak assignments were obtained for the highlighted residues):
KTDIDFELKETVKVIDGPFANFTGSIEEIDYDKSKVKVFVNMFGRETPVELEFTQIDKL

Figure S9: ^{1}H-^{15}N HMQC spectrum of 200 μM ^{15}N-labeled *Bs*NusG-CTD at 25°C. Backbone amide NMR signals assigned to specific residues are labeled with the corresponding residue type and number. Also shown is the amino acid sequence of *Bs*NusG-CTD and highlighting marks residues for which NMR peak assignment were obtained.

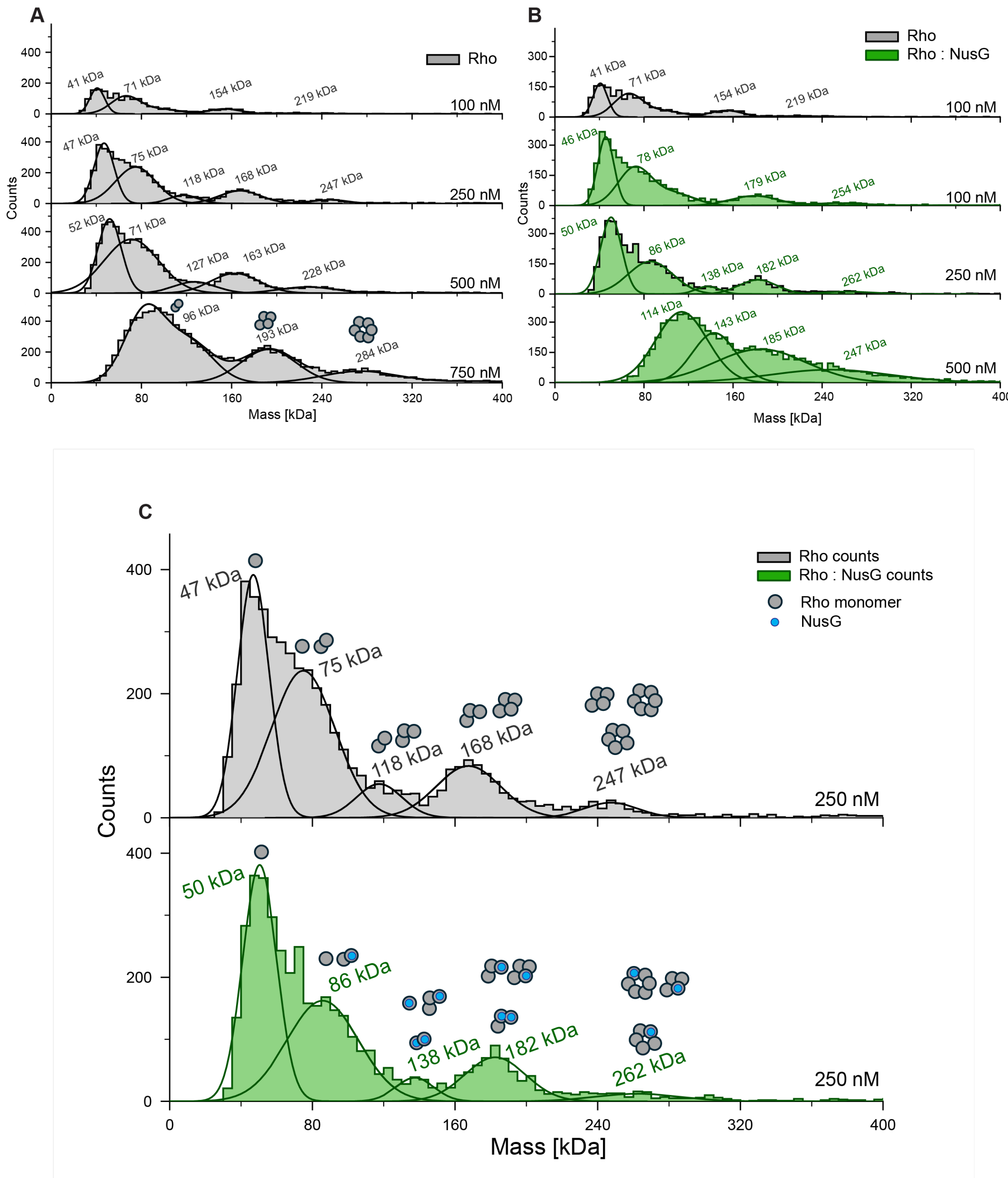

Figure S10: Mass photometry analyses indicate that *Bs*NusG associates with *Bs*Rho. Mass photometry data are shown for various concentrations of *Bs*Rho alone (gray) and *Bs*Rho with *Bs*NusG (green) at equimolar concentrations. (A) Vertical histograms graphs of *Bs*Rho alone (gray) shows that it populates various oligomeric states, although monomers dominate at concentrations between 100 nM – 500nM. At 750 nM, discrete peaks corresponding to dimer (96 kDa), tetramer (193 kDa) and hexamer (284 kDa) species are resolvable, suggesting that *Bs*Rho assembles via dimeric intermediates into hexamers. (B) Vertical histograms of *Bs*Rho alone (gray) at 100 nM and *Bs*Rho with *Bs*NusG at multiple concentrations (100 nM to 500 nM) show that there are no major mass shifts until 250 nM, with more pronounced shifts at 500 nM, thereby confirming concentration-dependent association of *Bs*NusG and *Bs*Rho. (C) Proposed mass assignments for mixtures of *Bs*Rho oligomeric complexes with *Bs*NusG at 250 nM (gray circles depict Rho

monomers and blue circles depict NusG). Due to the high heterogeneity of *Bs*Rho oligomer there are more than one mass combinations that is consistent with each resolved peak.

References Cited

1. Burgess RR. 2009. Protein precipitation techniques. Methods Enzymol 463:331–42.
